# Applications of Photoinduced Phenomena in Supramolecularly Arranged Phthalocyanine Derivatives: A Perspective

**DOI:** 10.3390/molecules25163742

**Published:** 2020-08-16

**Authors:** Simona Bettini, Ludovico Valli, Gabriele Giancane

**Affiliations:** 1 Department of Engineering of Innovation, University of Salento, Via per Monteroni, 73100 Lecce, Italy; simona.bettini@unisalento.it; 2 National Interuniversity Consortium for Materials Science and Technology, INSTM, Via Giuseppe Giusti, 9, 50121 Florence, Italy; gabriele.giancane@unisalento.it; 3 Department of Biological and Environmental Sciences and Technologies, University of Salento, Via per Monteroni, 73100 Lecce, Italy; 4 Department of Cultural Heritage, University of Salento, Via D. Birago, 64, 73100 Lecce, Italy

**Keywords:** sensors, fluorescence, time-resolved spectroscopy, UV-Vis, Charge-transfer, energy-transfer, supramolecular assembly, π-π interaction, hydrogen bonds, photocatalysis, solar cells, photodynamic therapy, fullerene, carbon allotropes, cyclodextrins, PDI, micelles

## Abstract

This review focuses on the description of several examples of supramolecular assemblies of phthalocyanine derivatives differently functionalized and interfaced with diverse kinds of chemical species for photo-induced phenomena applications. In fact, the role of different substituents was investigated in order to tune peculiar aggregates formation as well as, with the same aim, the possibility to interface these derivatives with other molecular species, as electron donor and acceptor, carbon allotropes, cyclodextrins, protein cages, drugs. Phthalocyanine photo-physical features are indeed really interesting and appealing but need to be preserved and optimized. Here, we highlight that the supramolecular approach is a versatile method to build up very complex and functional architectures. Further, the possibility to minimize the organization energy and to facilitate the spontaneous assembly of the molecules, in numerous examples, has been demonstrated to be more useful and performing than the covalent approach.

## 1. Introduction

The awareness that Nature brings into play disparate templates to fabricate complex macromolecules, such as proteins or DNA, has prompted scientists to investigate novel low-dimensional synthetic multi-molecular assemblies. Consequently, through the last decades remarkable amount of research has been dedicated to the preparation of bio-inspired hybrid supramolecular assemblies in consideration of their appealing and multifaceted applications in areas covering biology, chemistry, materials science, medicine, physics, from catalysis [1] to organic thin field-effect transistors [2], from cosmetics [3] to drugs [4], from imaging [5] to inks [3], from photovoltaic [6] to other energy-conversion devices [7], from sensing [8] to theranostics [9], from opto-electronics [10] to anticancer photodynamic therapy [11], from non-linear optics devices [12] to data storage systems [13]. Supramolecular assemblies can be described as hierarchical structures where each component is important to determine characteristics, employment and functionalities. So the idea of joining together different functionalities within the identical nanoassemblies has gained great attention [14]. Moreover, Ariga et al. have even introduced the concept of “nanoarchitectonics” [15].

In order to associate the diverse moieties of such multimodular structures, various approaches are available (used individually and independently or in combination) and, among them, the ones enabling thorough management of molecular architecture are the most employed: dispersion forces [16], electrostatic attractions [11], hydrogen-bonding interactions [17], hydrophobic effects [18], Langmuir-Blodgett [19] and Langmuir-Schäfer [20] techniques, Layer-by-Layer transfer [21], metal to ligand coordination [22], π-π stacking interactions [23], physical evaporation [24] and ultra-high vacuum methods [25], self-assembly [26], van der Waals interactions [27], and so on. These are some of the preferred means for the preparation of supramolecular and multimodular structures with tunable characteristics. In general such non-covalent interactions ensure the chance of generating stiff and distinct assemblies with anisotropic properties. In fact it is possible to modulate supramolecular assemblies structure through several parameters such as the central metal ion in the macrocycle [28], the construction methods [29], the ionic strength [30], pH [31], the presence of electro-donating or withdrawing functionalizations on the periphery [28], the solvent [32], the steric hindrance [33], the thermal treatments [34], etc. Such a novel approach allows, in several fields of applications of these hybrid structures, the prospects of amalgamation of multimodal procedures with the ultimate goal of accomplishing a synergistic effect [35].

Among the molecular building blocks for the fabrication of such structures, hefty tetrapyrrolic macrocycles, such as porphyrins, porphyrazines, phthalocyanines and other analogues (18 π -electrons aromatic macrocycles), play a role of primary importance due to their properties (rigidity, chance to deposit thin films, stability, extended conjugated π-electron cloud, intrinsic conductivity, light absorption capability with extinction coefficients of the order of magnitude of 10^5^ cm^−1^ M^−1^, long-lived fluorescence, redox activity, possibility to host uncoupled electrons on the central metal ion or in the aromatic cloud, rich electrochemistry, modulation of their characteristics through peripheral functionalization or variation of the central metal atom cation and so on). All these peculiarities allow to confer definite physico-chemical characteristics which are of paramount importance to manipulate the performance and interactions of these macrocycles as molecular Legos in supramolecular networks. The best instances of this engineering and regulation in supramolecular assemblies also this time come from biological networks where different components integrate to bring about indispensable and fundamental processes for living organisms.

**Scheme 1:**
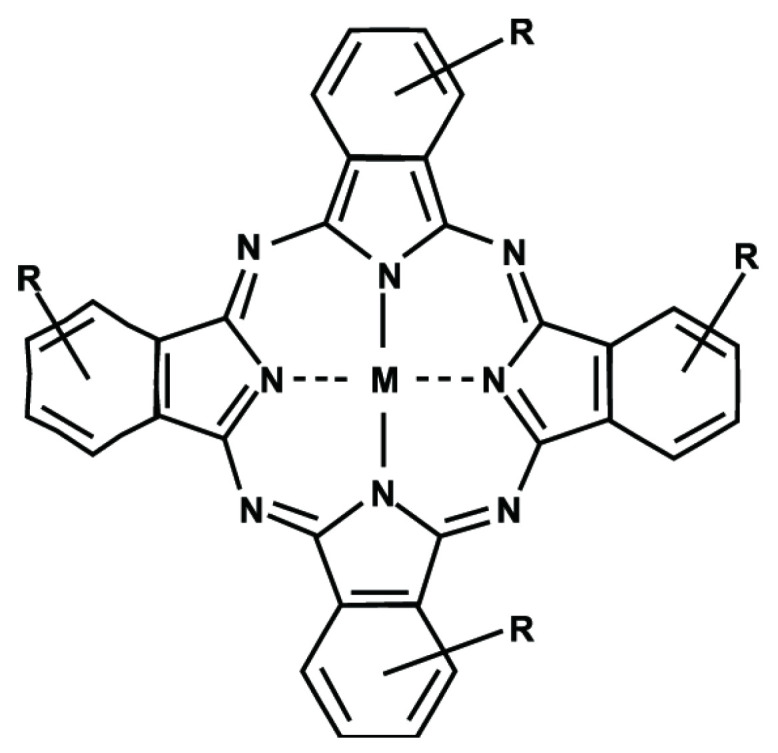
General structure of the phthalocyanine macroring, where M is the central metal ion or H_2_ in the case of the free base. R is the potential functionalization on the phthalocyanine periphery.

Herein, our review is focused on phthalocyanines (Scheme 1) and their applications in supramolecular assemblies in mainly four areas: charge and energy transfer, photocatalysis, photodynamic therapy and chemical sensors. The rationale of our choice resides in the more intense absorption of phthalocyanines above 550 nm (body tissues are moderately transparent in this wavelength range), superior photochemical and thermal stability in comparison with porphyrins [36].

Among compounds used in hybrid supramolecular structures in association with phthalocyanines, it is possible to cite antibodies [37], carbohydrates [38], carbon allotropes (fullerenes [39], graphene [40], nanotubes [7], etc.), cellulose nanocrystals [41], cucurbit[n]uril [42], cyclodextrins [43], DNA [44], liposomes [45], metal or oxide nanoparticles [46], peptides [47], perylene bisimides [48], polyaromatic compounds [49], polyoxometalates [50], protein cages [51], etc. All these substances in association with phthalocyanines in supramolecular structures permit to face the well-known tendency of these macroheterocycles to form large stacking aggregates—head to tail J type or face to face H-type stackings—(and consequently low-solubility, quenching of fluorescence and depletion of singlet dioxygen production in some areas of applications) and even offer the possibility to induce reversible conversion from aggregated to disassembled forms and vice-versa.

## 2. Pcs-Based Supramolecular Assemblies for Sensing Applications

**Figure 1:**
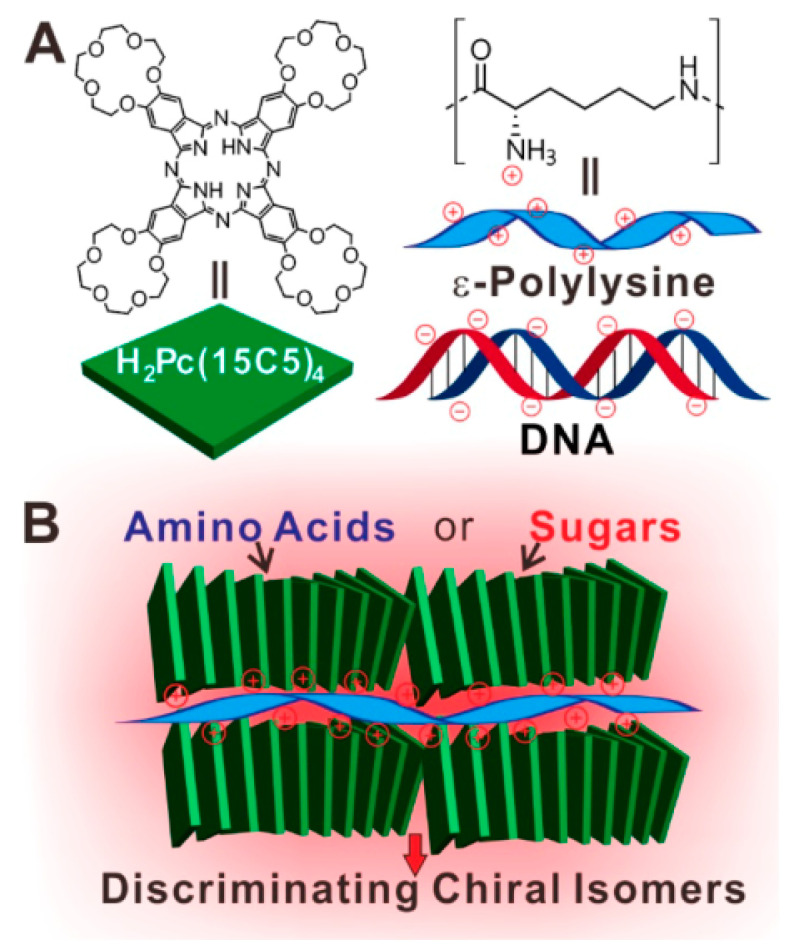
(**A**) Chemical structure of the tetrasubstituted 15-crown-5 ethers phthalocyanine derivative and the two biomolecules. (**B**) Schematic representation of the formation of Pc/poly(l-lysine) assemblies. Reprinted with permission from ref. [52]. Copyright 2017 American Chemical Society.

In an interesting paper [52], Jiang and collaborators have proposed the unusual realization of an assembly capable of identifying different chiral analytes, such as amino acids and sugars. The strategy has been to couple a tetrapyrrolic moiety, such as a phthalocyanine derivative, with a biomacromolecule; the first one bears four 15-crown-5 ethers (Figure 1A) which coassemblies with two biomacromolecules, a negatively charged DNA or poly(l-lysine). The crown-ether has a dual function: to induce amphiphilic properties and pliability to the macrocycle, while the other two components have to relocate optical activity to the co-assemblies. Such supramolecular assemblies have been fabricated by both the Langmuir-Blodgett (LB) [53,54] and the Langmuir-Schäfer (LS) techniques [52] since the Coulombic interaction between species with opposite charges allows the transfer of robust dyads; in this case with the presence of the amphiphilic phthalocyanine floating on the water surface and DNA or poly(l-lysine) dissolved in the subphase. The surface pressure vs. area per molecule curves already gave indications of the interaction at the air-water interface between the biomacromolecules and the phthalocyanine. The UV-Vis spectra of the LS films of the pure phthalocyanine evidence an unequivocal hypsochromic shift suggesting the presence of H-aggregates; such a shift becomes even larger when poly(l-lysine) is inserted in the LS films. Next also circular dichroism (CD) investigations were carried out; it is worth to observe that, even though the phthalocyanine is not optically active, the fabricated LS films evidence CD signals. The generation of supramolecular chiral assemblies has been also checked in LS films transferred with DNA and poly(l-lysine). In particular in this last case a positive Cotton effect has been discovered in the Q-band; but it is absolutely discrepant from that observed for the pure phthalocyanine film thus suggesting a modification of the supramolecular architecture of the composite multilayer. The positive charges in poly(l-lysine) corroborate the effective interaction existing with the phthalocyanine even at the air-water interface. Further AFM, XRD and FT-IR investigations [52] confirmed the origination of a weak chirality when films of the pure phthalocyanine were deposited; but, on the other side, the presence of poly(l-lysine) induces the handover of biomaterials chirality to the phthalocyanine coassemblies. 

Moreover the composite poly(l-lysine)/phthalocyanine multilayers display good fluorescence characteristics upon irradiation at 470 nm suggesting that the polypeptide inhibits macrocycle dense packing and so also fluorescence quenching. The most remarkable result is the observation that the interaction of these films with amino acids in aqueous media permits to detect not only the amino acids themselves but even to distinguish enantiomers of several of them. In the case of acidic amino acids (aspartic and glutamic acids and proline) the ability to discriminate between the corresponding l- and d-enantiomers has been exhibited: in fact, above all in the instances of glutamic and aspartic acids, the l-enantiomers provoke a notable fluorescence enhancement, while the d-enantiomers an evident contraction. In the case of proline the phenomenon is less pronounced, but always observable.

Otherwise, basic amino acids do not influence fluorescence; the most immediate rationale is that their interaction with poly(l-lysine) is really faint. Different is the case of aromatic amino acids, such as phenylalanine and histidine, since a reduction of fluorescence has been observed for both stereoisomers. This observation has prompted the investigation of the corresponding fluorescence quenching rate: it has been observed that d-phenylalanine fastens fluorescence quenching in comparison with the slower phenomenon detected in the case of l-phenylalanine. The rationale is that the aromatic amino acid propagation within the composite films depends on the chirality.

Another consideration is that the significant presence of hydroxyl groups in carbohydrate molecules potentially could ensure the thorough interaction with the phthalocyanine/poly(l-lysine) composite films and thus induce changes in the fluorescence of the macrocycle. d-galactose, d-glucose and d-mannose were used in aqueous solutions and the immersion of the LS multilayers has provoked a remarkable quenching of fluorescence, even though their discrimination by steady-state fluorescence is not possible. Hence the same approach already described for the aromatic amino acids has been employed; and in fact the investigation of the fluorescence quenching rate allows distinguishing D-glucose with the fastest rate from d-galactose and d-mannose which has the slowest rate.

Figure 1B illustrates the rationale given by the authors about the possible mechanism involved in the sensing discrimination of enantiomers. In the case of D- and L-glutamic acid (a molecule which doesn’t exhibit peculiar photophysical characteristics) it appears straightforward to propose that the fluorescence variations directly derives from changes in macrocycle aggregation. It has been suggested that D-glutamic acid could induce a more significant aggregation of the phthalocyanine molecules; in contrast, L-glutamic acid molecule contribute to slightly move away the macroring molecules. Further analysis by AFM, CD, and UV-Vis spectroscopy are consistent with these conclusions [52].

In a paper by Zhu et al. the possibility to detect nucleic acids was investigated above all considering the usual approach already employed for their revelation (addition of fluorescent probes) [55]. In fact the fluorescence of nucleic acids is poor and can be enhanced through the interaction with peculiar dyes. The work proposed by C. Zhu et al. was stimulated by a research by Gurrieri et al. [56] who explored the supramolecular aggregates of an anionic porphyrin derivative with polylysine for the recognition of DNA. 

Zhu et al. have used tetracarboxyaluminum phthalocyanine (AlC_4_PC) which displays intense fluorescence in the NIR wavelength range; the presence of the negative charges permits thorough interaction with polylysine (a polycationic polypeptide) with a pKa of 9.9, thus inducing also absorption (at 686 nm) and fluorescence (at 701 nm) suppression. But the most important observation is that upon the successive interaction with nucleic acids the former fluorescence is retrieved. This analysis has prompted the suggestion of this approach for the realization of chemical sensors of nucleic acids with fluorescence excitation and emission in the NIR range in order to preserve biomaterials from more energetic irradiation. The active layers of these sensors have exhibited excellent responsiveness, speed and stability.

During the investigations also the influence of pH has been controlled; best results were obtained buffering the system at pH = 6.73 by hexamethylenetetramine–HCl buffer solutions. As a further control, also the effect of the AlC_4_Pc/polylysine concentration ratio was examined; the best performances were observed when both concentrations were fixed at 10^−7^ M.

Another straightforward observation concerned the effect of ionic strength whose value obviously influences the AlC_4_Pc/polylysine interaction considering its electrostatic origin. In the case of sodium chloride the upper limit for the salt concentration is 20 mM.

Finally, as far as selectivity is concerned, the data from investigations are summed up in Table 1.

Achadu and Nyokong have proposed functional composites of graphene quantum dots (GQDs) and Co(II) pyrene-substituted phthalocyanine (CoPc) as selective and sensitive moieties for cyanide ions (CN^-^), a severely harmful anion [57]. GQDs have been extensively used because of their biocompatibility and photoluminescence properties and since their π-electron cloud and surface pendant groups allow functionalization by other materials. GQDs’ nanoconjugates with tetrapyrrolic macrocycles were already used by the authors in other studies and GQDs’ fluorescence was quenched by phthalocyanines and porphyrins. Cyanide ions have been demonstrated the only ones able to switch on the fluorescence of GQDs.

The first investigations concerned GQDs (synthesized by a hydrothermal procedure) whose emission, upon irradiation at 340 nm, was observed at 445 nm with a 0.31 quantum yield and a 5.7 ns lifetime.

The GQDs-CoPc composites have been prepared by addition of GQDs to CoPc [57]; a bathochromic shift by 10 nm of the phthalocyanine Q band has been registered, from 706 to 716 nm, while the pattern of the spectrum was remarkably influenced by GQDs thus suggesting the thorough interaction between the dots and the macrocycles.

Studies by transmission electron microscopy (TEM) have evidenced another typical behavior: the generation of phthalocyanines π-π aggregates onto GQDs. Moreover the generation of the GQDs-CoPc hybrids is accompanied by the fluorescence “turn OFF” of GQDs with a prompt suggestion of an energy transfer in a donor-acceptor dyad but the most important following discovery is that fluorescence is restored by interaction of the composite material with cyanide ions, CN^−^. The dependence of the fluorescence intensity on CN^−^ concentration is linear in the interval 0–50 nM and can be expressed by the following Equation (1):

(1) $$\frac{\Delta F}{F_{0}}=1+K\left[{\mathrm{CN}}^{-}\right]$$

where *F* and *F*_0_ are the fluorescence intensities in the presence and absence of CN^−^ ions and *K* is the fluorescence constant. The limit of detection for cyanide ions has been calculated to be 0.5 nM.

**Figure 2:**
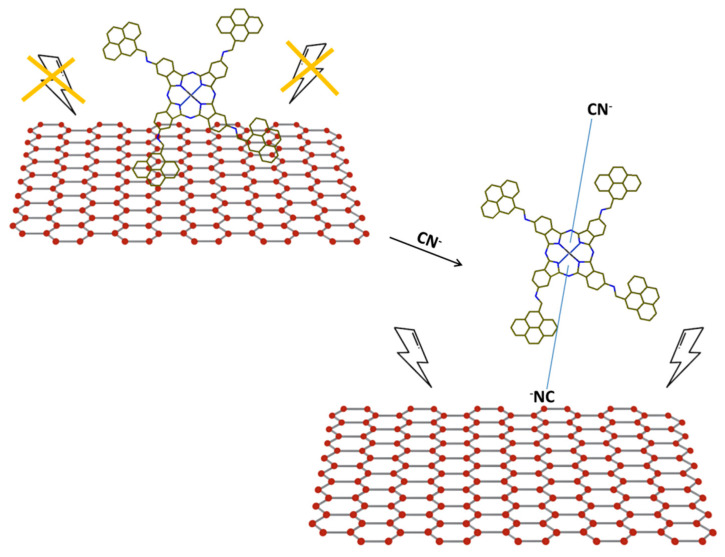
Scheme of the CN^−^ sensing mechanism by supramolecular assembly GQDs-CoPc proposed in the Ref [57].

The proposed rationale (Figure 2) for the interaction among the cyanide ions and the GQDs-CoPc hybrids entails the binding of the CN^−^ ion to cobalt central metal atom thus spacing GQDs and CoPcs out. This separation remarkably hinders the energy transfer and consequently reawakens the fluorescence of GQDs. The gradually enhanced bathochromic shift of the CoPc Q-band in UV-Vis absorption spectroscopy to a greater extent supports the axial ligation of cyanide ions to the Cobalt atoms.

In order to check the selectivity of the GQDs-CoPc hybrids towards the cyanide ion a long series of anions have been experimented: F^−^, Cl^−^, Br^−^, I^−^, H_2_PO_4_^−^, HSO_4_^−^, NO_3_^−^, ClO_4_^−^, SCN^−^, CH_3_COO^−^, HSO_3_^−^, SO_4_^2−^ and CO_3_^2−^; their perturbation to the response to cyanide ion is conspicuously lower as an additional confirmation of the chemical sensor selectivity [57].

## 3. Pcs-Based Supramolecular Assemblies for Photovoltaic Applications

Unlike porphyrins, tetrapyrrolic macrocycles often associated to phthalocyanines for their chemical and physical features [58,59] and for the functionalization approaches usually employed [60], Pcs show both electron donor and electron acceptor characteristics depending on the chemical species they are interfaced. Further, photoinduced phenomena can be tuned changing the phthalocyanine central metal atom [61,62].

**Figure 3:**
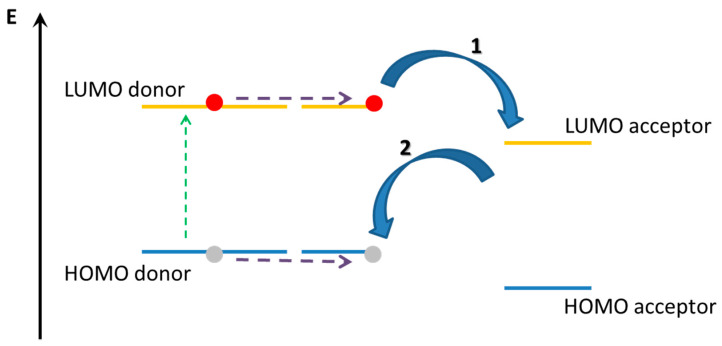
Energy levels of electron donor and electron acceptor during the electron-hole couple formation and separation.

In this section, supramolecularly assembled dyads formed by phthalocyanines acting as electron donor compounds will be considered and examined. In fact, the relevant light harvesting of Pcs derivatives, even higher than that one of porphyrin derivatives, ensures an efficient electron excitation under visible illumination and, if coupled with suitable molecules, efficient electron donor features. Charge transfer mechanism in conjugated compounds is strongly limited by the binding energy of the lowest singlet excitation (about 0.4 eV) [63,64]. In this class of organic compound, the photo-generated exciton is a thermodynamically stable specie, so the charge separation (CS) step appears forbidden or not-favored. On the contrary, the charge transfer (CT) process can be obtained using electron donor and electron acceptor species in both multi-layered and blended geometries. In this approach, photons are absorbed by the donor and/or acceptor species. In the case that most of irradiating photons are absorbed by the electron donor species, an electron-hole couple is formed on this compound as a consequence of the promotion of π-electrons from the HOMO to the LUMO level (Figure 3). In order to separate the hole-electron pair, the exciton has to move up to reach the interface with the acceptor element [65].

Then, the electron promoted in the LUMO level of the donor element is transferred on the LUMO level of the acceptor compound, leaving the hole in the HOMO level of the donor (the charge separation process is indicated with the arrow labeled as **1**). This process, known as photon-induced electron-transfer, converts light into electric charges. Obviously, in order to obtain an electric current is mandatory to overcome the Coulombic attractive force and to ensure an efficient migration of the separated charges toward the two electrodes. Furthermore, it is necessary that the species obtained after the charge separation step has the lowest energy configuration than any intermolecular excited state. Again, as highlighted in Figure 3, the recombination charge process (represented by the arrow labeled as **2** in Figure 3) competes with the charge separation and the equilibrium between these two mechanisms is strongly influenced by the molecular parameters. In this context, as well as the deposition techniques and parameters, the self-assembly of donor-donor and donor-acceptor molecules represents a successful approach to maximize the charge separation process.

Hierarchical organization across all length scales is considered a relatively simple approach able to mimic the natural photosynthesis. Supramolecular chemistry, in fact, allows to build up extremely complex structures bringing multifaceted features [66].

For example, the effect of the relative position between a free-based phthalocyanine (H_2_Pc) and a perylene bisimide (PDI) on the charge separation and charge recombination was considered. The electronic coupling, which is particularly influenced by the molecular disorder, was demonstrated to exponentially decrease when the intermolecular distance increases [65]. The role of the molecular orientation was evaluated and it was concluded that the use of planar structures can be detrimental if compared to spherical (or pseudo-spherical) structures like C_60_.

**Figure 4:**
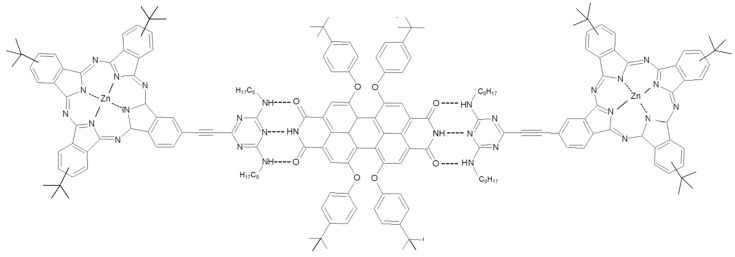
Chemical structure of Pc and PDI reported in the reference [69].

Nevertheless, the appropriate energetic levels of Pcs and PDI prompted Guldi’s and Torres’ research teams to propose several photo-active system based on the Pcs-PDI dyad. The electronic communication between Pcs and PDI was already used to carry out TiO_2_-based dye sensitized solar devices and Forster resonant energy transfer from PDI to Pcs was used to enhance the spectral range of photon absorption up to blue region of the visible spectrum [67,68]. Starting from these evidences, a supramolecular three-dimensional Pcs (in the role of electron donor)–PDI (as electron acceptor) photo-active layer was built up [69]. In particular, a Pc functionalized with a ditopic melanine moiety supramolecularly binds a bifunctional PDI by means of hydrogen bonds (Figure 4). As highlighted by UV-Vis and fluorescence measurements, a complex formed by two Pcs and one PDI derivative linked by hydrogen bonds is obtained. 

**Figure 5:**
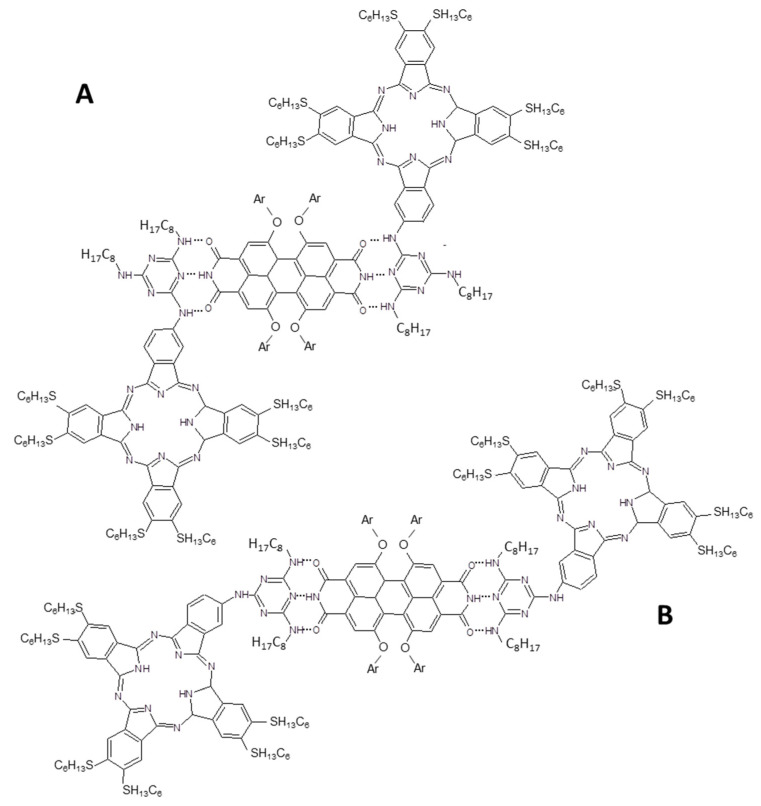
Melanine substituted Pc used to form the supramolecular assembly with functionalized PDI derivative [71]. In (**A**,**B**) the two possible supramolecular configurations are reported according to the authors’ rationale [71].

Similarly a molecular assembly was obtained by the same authors by linking a ruthenium metallated Pc with a functionalized PDI by means of covalent binding [70]. Even in this case, the supramolecular approach appears more versatile since it allows to modulate the donor:acceptor ratio and to deeper investigate the photon-induced communication between the two species. Nevertheless, in both approaches, as reported by the authors, it was not possible to evidence any charge transfer reaction from PCs and PDIs. This issue was overcome by the modification of the PCs substituents [71]. In particular, the phthalocyanine (a free-base in this contribution) is still asymmetrically substituted with a melanine unit, but the methyl group is now substituted with an alkanethiole on the Pc periphery (Figure 5).

Two different configurations can be theoretically adopted by the supramolecular adduct made by two Pcs and a PDI derivative but the authors highlighted that type A in Figure 5 is favored. The supramolecular adduct, driven by hydrogen bonds, was characterized by ^1^H-NMR, steady-state and transient absorption spectroscopy. It is very interesting that no charge and/or energy transfer is observed when the PCs units are excited to their absorption maximum, on the contrary when PDI moiety is opportunely excited a thermodynamically favored charge separation is induced from PDI to Pc derivatives. The excited electron on the LUMO level of PDI leaves a hole in the valence band that is filled by an electron from Pc and a charge separated state with a lifetime of about 8 ns was measured by laser flash photolysis. 

**Figure 6:**
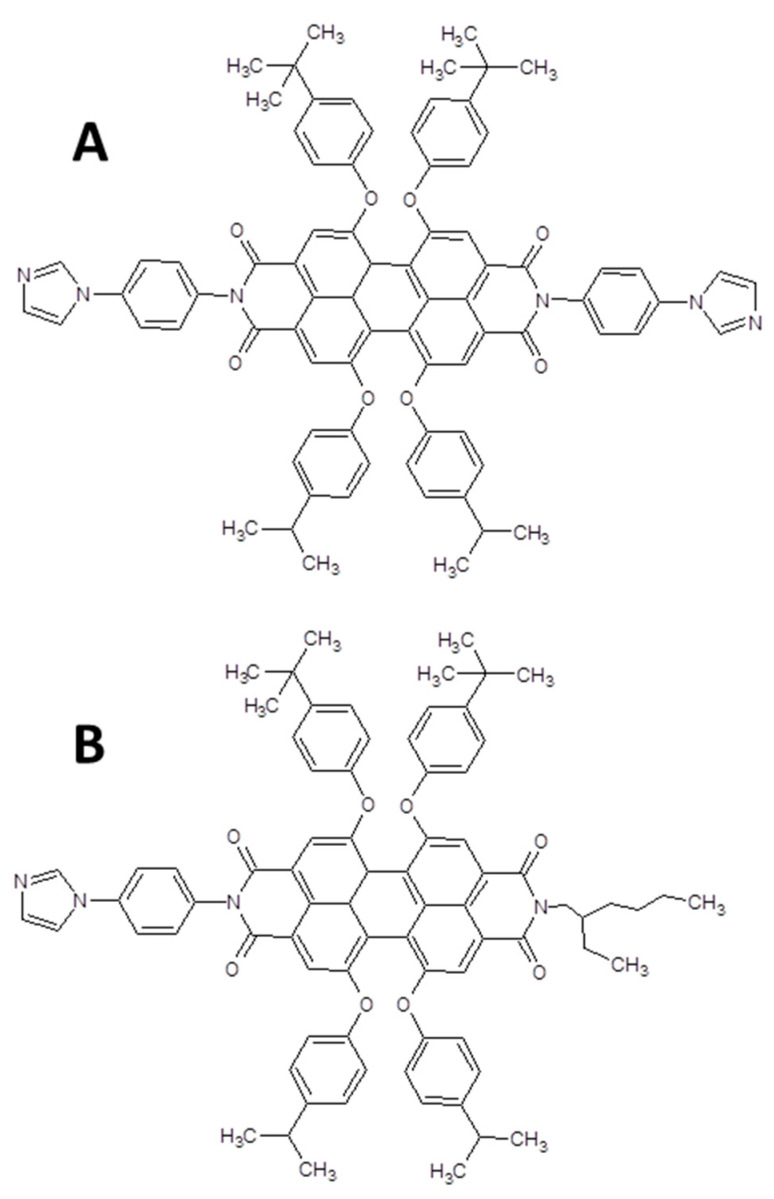
Chemical structures of the bis-(**A**) and mono (**B**) substituted PDIs used to form the supramolecular adducts with the ZnPc reported in the reference [72].

Two systems represented by PDI derivatives/ZnPc substituted were characterized by Sastre, Fukuzumi and collaborators. The authors used a different approach to obtain two supramolecular adducts: a dyad formed by the octaepoxy substituted ZnPcs and an imidazolyl bis- and mono-substituted perylene bisimide, reported in Figure 6A,B, respectively [72].

The imidazole substituents interact with the central metal atom inducing the formation of the supramolecular adducts. The formation of a ZnPc/PDI B and a triad PDI A/ZnPc/PDI A was demonstrated and confirmed by means of UV-Vis and fluorescence titration; furthermore ^1^H-NMR suggests a stable enough formation of the dyad and triad and lifetimes of the separated charge state of about 9.8 ns for the triad and about 3 ns for the supramolecular dyad. 

**Figure 7:**
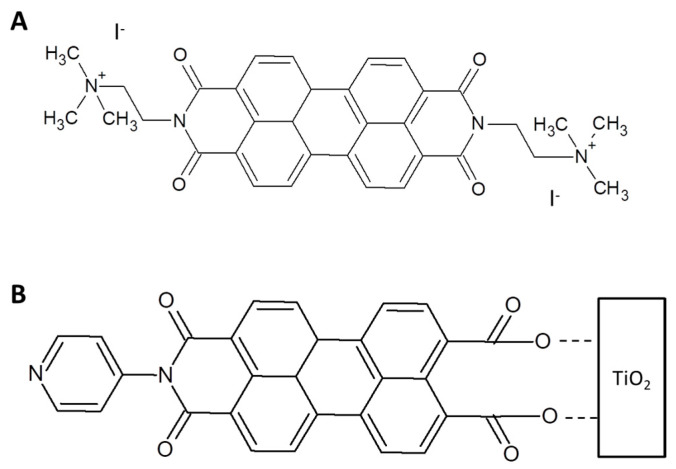
(**A**) Double charged PDI derivative used to form the photoactive dyad with ZnPc. (**B**) Asymmetrically substituted perylene imide linked to TiO_2_ to build up a dye sensitized solar cell [76].

Even electrostatic interaction coupled with π-π stacking allows to build up supramolecular assemblies [73,74]. A supramolecular dyad between a tetrasulfonated Zn phthalocyanine and a perylene dimide derivative reported in Figure 7A is formed in [48]. The authors evidenced the formation of well-known elongated 1D structures of perylene derivatives [21,75] that were affected when PDI is dissolved in water in presence of the sulfonated porphyrin. DLS analysis suggests that large aggregates of PDI/Pcs are formed under the action of π-π and ionic interactions. Fluorescence and UV-Vis studies of PDI solutions in presence of increasing concentration of the anionic ZnPc derivative were used to determinate the binding constant (about 3 × 10^4^ M^−1^) and to evidence the energy transfer process from ZnPcs to the singlet-excited state of PDI. The electron transfer between the two species was further confirmed by electrochemical measurements. 

Asymmetrically substituted perylene imide (PyPMI) (Figure 7B) was used to simultaneously supramolecularly bind a ZnPc and TiO_2_ to build up a dye sensitized solar cell [76]. Pyridine substituent interacts with the Zn atom of Pc derivative and the anhydride termination was used to anchor the supramolecular ZnPc/PyPMI dyad on the TiO_2_ surface. Even though the UV-Vis spectrum of the dyad is a linear combination of the UV-Vis spectra of the two dyes, the IPCE% of the ZnPc/perylene imide dyad is 40% higher than the sum of the devices obtained using ZnPc and perylene imide separately. The authors proposed that an electron transfer is induced from PyPMI to TiO_2_. On the other side, when ZnPc is excited the electron is transferred on PyPMI and it is followed by a charge transfer on TiO_2_. It allows to increase the charge separation (localized on ZnPc and TiO_2_) and to maximize all the cell parameters. 

Anyway, the most used electron acceptors coupled with Pcs belong to the fullerene family. In fact, the chemical configuration of C_60_ and C_70_ allows to these compounds to accept up to six electrons [77], minimizing the reorganization energy. Not functionalized C_60_ and C_70_ were demonstrated to be able to form supramolecular adducts with tetra-butyl Pc in toluene by means of dipole-dipole interaction. Energy transfer was theoretically predicted and experimentally demonstrated by transient absorption spectroscopy [78]. Same functionalization was added on a Zn metallated Pc and the interaction with C_60_ and C_70_ was evaluated in toluene and dichlorobenzene, confirming the results previously produced [79]. Both static and dynamic quenching were observed in presence of C_60_ and C_70_. Higher binding constant was measured for the complex C_70_/ZnPc and the proposed mechanism for the formation of the supramolecular adduct was the electrostatic interaction between the electron clouds of the two compounds. Ab initio calculation was used to evidence that, in the ground state, the HOMO level is located on Pc while the LUMO level is placed on the fullerene moiety of the complex. Furthermore, theoretical calculation of formation heat suggested that the formation of C_70_/ZnPc is more favored than the supramolecular complex C_60_/ZnPc (about −1.3 kcal mol^−1^ and −0.6 kcal mol^−1^ for C_70_ and C_60_, respectively). In any case, theoretical simulations were not able to predict the photoinduced charge transfer and the energy transfer appears energetically favored. Size selection in the formation of non-covalent complexes with C_60_ and C_70_ was achieved inserting long alkyl chains on the Pc periphery [80]. In particular, eight octyloxy groups attached to the Pc periphery (2,3,9,10,16,17,23,24-octakis(octyloxy)-29H,31H-phthalocyanine) were used to form a branched structure. The authors report that the supramolecular adduct is formed both in presence of C_60_ and in presence of C_70_, even though NMR in toluene highlighted that a larger binding constant is obtained for the supramolecular adduct obtained with C_70_. Theoretical studies suggest that the supramolecular adduct C_70_/ZnPc is formed in the so-called side-o configuration with a strong involvement of the substituents in the adduct formation. 

**Figure 8:**
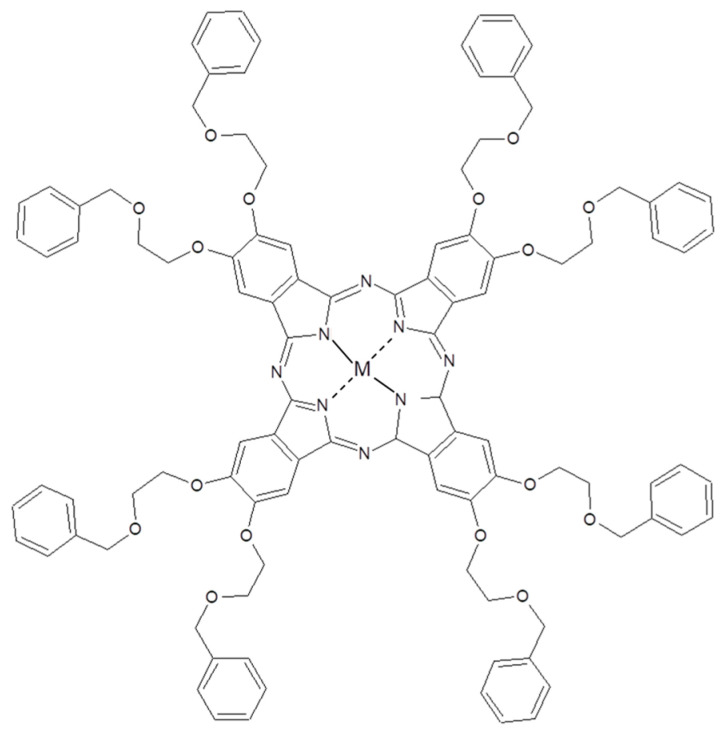
Chemical structure of (2,3,9,10,16,17,23,24-octakis(octyloxy))-29*H*,31*H*-phthalocyanine used in [81].

A similar approach was used to build-up a Pc/fullerene supramolecular adduct using an octopus-like metallated phthalocyanine functionalization (Figure 8) [81]. Even in this case, a higher affinity was observed towards C_70_ than C_60_ both by means of theoretical and experimental proofs [81]. 

From an applicative point of view, the use of not functionalized C_60_ and C_70_ presents the main drawback of the self-aggregation phenomenon. Two possible approaches are mainly used to minimize the self-aggregation of fullerene: 1) the functionalization of the bucky-ball carbon allotrope, 2) the use of chemical compounds, such as polymers, to disperse them. The last approach was proposed by Jurow and co-workers in [82]. The authors functionalized a commercial fluorurated zinc phthalocyanine with thioalkane e thiofluoroalkane group. The presence of florurated substituents enhances the interaction of Pcs with C_60_ inducing the formation of supramolecular nanostructures [83]. The ratio C_60_/Pc is crucial, in this case, to preserve the supramolecular formation and the uniform covering of ITO (and glasses) substrate when the supramolecular assemblies are transferred by the electrode immersion method. 

**Figure 9:**
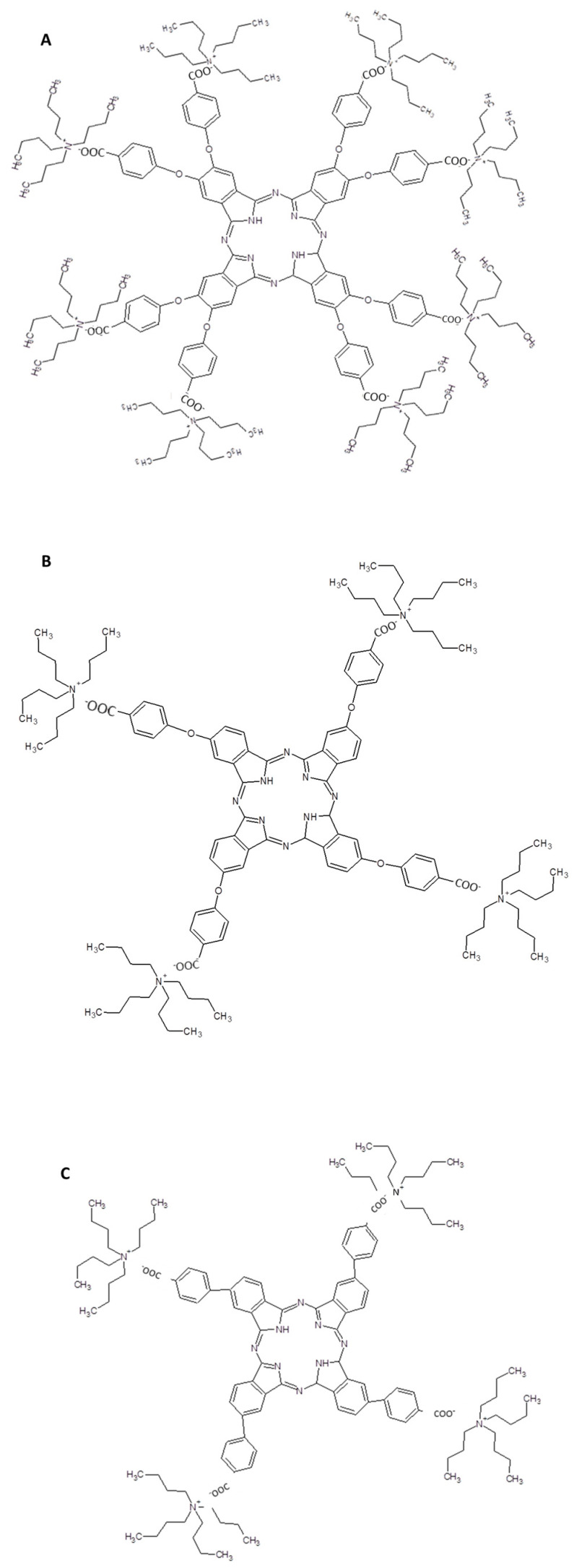
Chemical structures of the three carboxylated phthalocyanines used to form the electrostatically bounded supramolecular dyads with Li^+^@C_60_ [84]. (**A**–**C**) are the three phthalocyanine derivatives used to form complexes with fullerenes.

Inserting a lithium cation into the C_60_ cage (Li^+^@C_60_) was possible to assemble a supramolecular adduct with three carboxylated phthalocyanines (Figure 9) [84]. 

The supramolecular assembly in dimethylsulfoxide:benzonitrile (*v*/*v*) = 1:1 was studied by means of spectroscopic techniques. For the Pcs in Figure 9B,C a complex formed by 2 fullerenes and a Pc is obtained and the binding constants were estimated about 10^12^ M^−2^ for both of them. When Pc in Figure 9A is used, a complex formed by one fullerene derivative and one phthalocyanines is obtained and the binding constant appears strongly reduced. Photoinduced charge transfer from functionalized Pcs B and C and (Li^+^@C_60_)_2_ was observed and important parameters, such as reorganization energy and electronic coupling, were estimated. Then, Pcs/(Li^+^@C_60_)_2_ assemblies were transferred on an optically transparent electrode by electrochemical method. High incident photon to converted electron (IPCE) values indicate that an intense photocurrent takes place after the light irradiation. 

**Figure 10:**
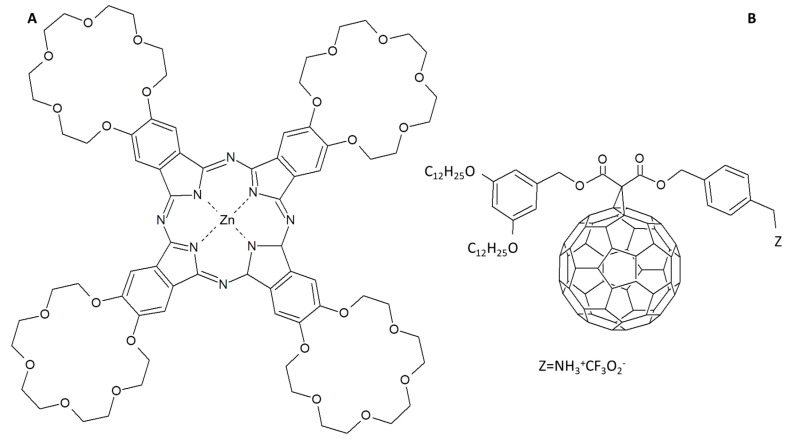
(**A**) 18-crown tetra functionalized ZnPc and (**B**) C_60_ derivative chemical structures [85].

Another example of dendritic substituted Pc is shown in [85] where a donor/acceptor supramolecular dyad is obtained coupling a ZnPc functionalized with 18-crown substituents and a C_60_ derivative bearing an ammonium head group (Figure 10). The −NH_3_^+^ group is crucial to prevent the formation of large aggregates of C_60_ as well as the crown substituents limit the aggregation of Pc molecules. The authors demonstrated that a 1:1 ratio of C_60_/Pc is supramolecularly formed by means of π-π interactions, charge transfer and crown substituents that play a cooperative role in the supramolecular assembling. In fact, increasing the numbers of crown-substituents on the Pcs periphery, C_60_/Pc assembly is formed bearing one fullerene and four phthalocyanine derivatives. 

**Figure 11:**
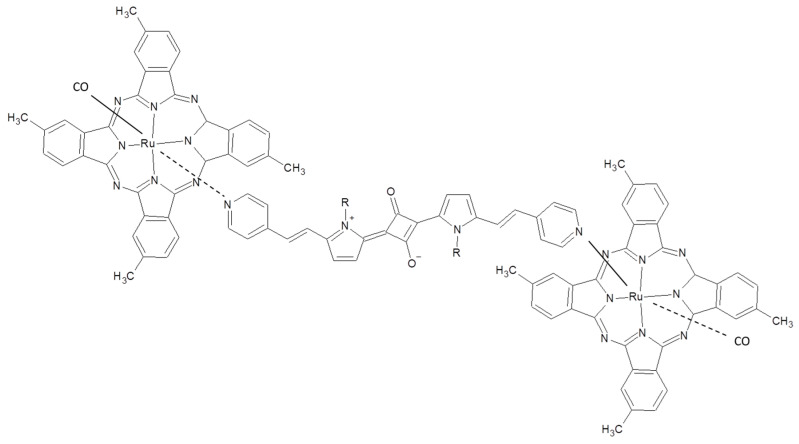
RuPc/squaraine derivative/RuPc photoactive supramolecular assembly reported in [86].

In the supramolecular dyad represented by RuPc/squaraine derivative/RuPc reported in Figure 11, a photoinduced intramolecular charge transfer process within the fragments of the assembly was observed in toluene, THF, and dichloro-methane. In particular, by means of photophysical studies, the authors evidenced the formation of radical ion pair state RuPc-squaraine^•−^-RuPc^•+^ with a very long lifetime (24 µs) [86]. When the supramolecular assembly is blended with the well-known PCBM in a ratio 1:4 a solar cell with a relatively low efficiency of about 0.3% under solar simulator irradiation was obtained. 

The role of induced order by self-assembled Pcs in a blend with PCBM was investigated in [87]. It was demonstrated that the supramolecular preferential arrangement induced by a fluoride atom attached to the Pc central gallium atom increases the PCE from a very low 0.03% to 0.4% (in the first case a −OH is linked to the Ga central atom).

**Figure 12:**
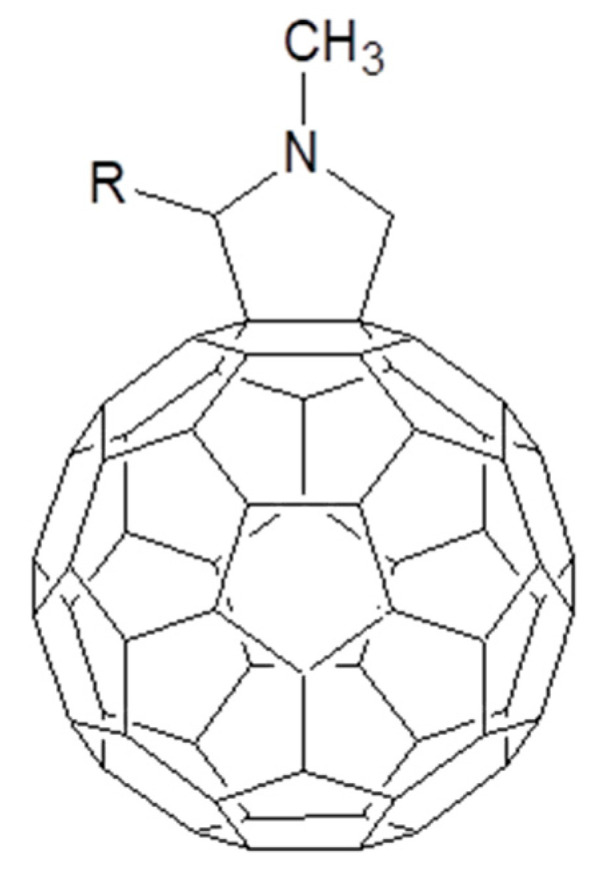
C_60_ fulleropyrrolidine chemical structure.

Moreover, the highest number of contributions about the formation of supramolecular aggregates of phthalocyanines and C_60_ derivatives is related to the use of the well-known fulleropyrrolidines [88]. In Figure 12, C_60_ fulleropyrrolidine chemical structure is reported. 

Before examining the large number of fulleropyrrolidines used to promote supramolecular assemblies with Pcs for photoinduced charge transfer and charge separation, it is useful to consider a contribution by Sariciftci and collaborators that clearly evidences the role of pyridine functionalization on C_60_ and how it influences the photovoltaic response of a device using it as an electron acceptor [89]. In particular, the performances of a solar cell where a ZnPc and 3-pyridyl functionalized fulleropyrrolidine were compared with those ones of an equivalent device where the PCBM was used as electron acceptor. The pyridyl substituent is able to complex the Zn atom of the ZnPc as testified by the enhancement of solubility of the metallated phthalocyanine in dichloromethane. Then, the authors transferred a layer of ZnPc, by means of thermal evaporation, on the top of an ITO/PEDOT:PSS substrate. In order to compare the performance of PCBM and pyridyl functionalized C_60_, the two fullerenes derivatives were spun on the top of ZnPc layer. All the cell parameters (short circuit current, open circuit voltage and fill factor) are higher for the solar cell based on the pyridyl functionalized C_60_ [90]. It is a consequence of the high π-electrons overlap of the donor and acceptor species obtained when the supramolecular adduct is formed. Furthermore, the higher solubility of the fulleropyrrolidine/ZnPc complex leads to a higher interface between the donor and acceptor species. 

The photodevice efficiency was further enhanced, from 1.5% to 2% under 1.5 AM solar simulator illumination, when a blend of the same fullerene derivative and the organic polymer poly((2-methoxy-5-(3,7-dimethyloctyloxy)-*p*-phenylene)vinylene (briefly named MDMO-PPV) is used [91]. The authors evidenced that a very small amount of fulleropyrrolidine mixed with PCBM (2% and 98%, respectively) is enough to double the device efficiency in presence of MDMO-PPV. 

**Figure 13:**
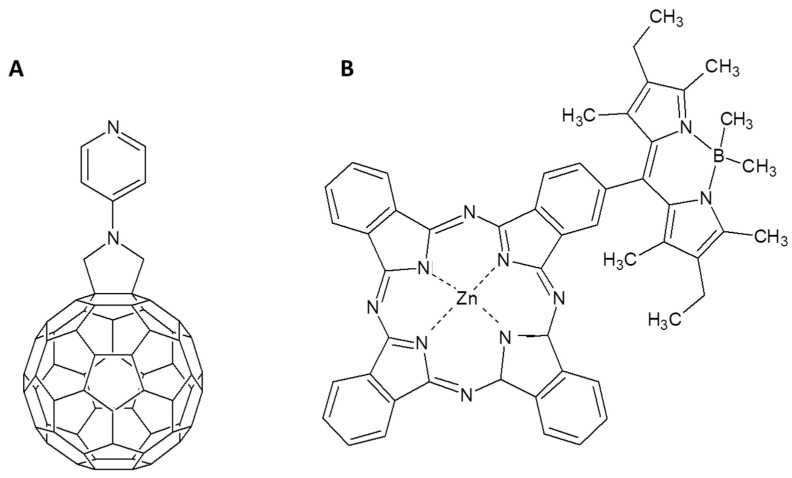
(**A**) Pyridine functionalized fulleropyrrolidine and (**B**) BODIPY-substituted ZnPc proposed in [92].

The interaction between the pyridyl substituent and the zinc central atom of the phthalocyanine was largely used to form photoactive dyads and triads. For example, a supramolecular assembly between the C_60_ derivative in Figure 13A and the ZnPc in Figure 13B was obtained by means of the pyridine-Zn atom interaction [92]. The ZnPc is covalently functionalized with a BODIPY (4,4-difluoro-4-bora-3a,4a-diaza-s-indacene) able to transfer energy, after appropriate irradiation, to the ZnPc core. It allows to extend the photon absorption spectrum to almost the complete visible range. So, the BODIPY acts as energy donor toward the ZnPc that works as energy acceptor. When the C_60_ derivative supramolecularly binds the BODIPY functionalized ZnPc, the photoexcitation of BODIPY is immediately followed by an energy transfer on ZnPc moiety and then by the formation of BODIPY-ZnPc^•+^/C60^•−^ radical-ion pair state. 

A remarkable achievement is the charge separated state lifetime that reaches 40 ns in toluene, 8 times longer than the system C_60_/ZnPc without the BODIPY moiety. This enhancement was explained by the authors as a consequence of the anodic shift of the oxidation potential of the ZnPc when linked to the BODIPY. This phenomenon induces a shift of the driving force of charge recombination into the Marcus inverted region, retarding the radical anion recombination. Furthermore, attaching a porphyrin to a BODIPY it was possible to obtain an antenna complex able to capture light and to efficiently transfer energy on ZnPc [93]. In presence of a phenylimidazole-functionalized fulleropyrrolidine the electron is transferred from Pc to C_60_ derivatives that is supramolecularly linked to the Zn atom of Pc.

The inclusion of one or two corannulene functionalizations was another strategy used by the same authors to increase the charge separation lifetime [94]. The hydrophobic nature of corannulene stabilizes the charged state in polar solvents and improves the delocalization of the charges in one-electron oxidized ZnPc by means of π–electron cloud extension. 

Another method to enhance the charge separated state lifetime is to increase the distance between the two species of the supramolecularly arranged Pc-fullerene derivative assembly. The lifetime of the charge separated state in a supramolecular dyad C_60_/ZnPc was increased from 2.5 ns up to 4.8 ns simply using a phenylimidazole functionalization in place of a pyridine on a fulleropyrrolidine [95]. In the sake of truth, the functionalization influences the assembly formation as evidenced by the different binding constant estimated for pyridine- and phenylimidazole- functionalized fulleropyrrolidine (10^4^ and 10^5^ M^−1^ respectively). 

**Figure 14:**
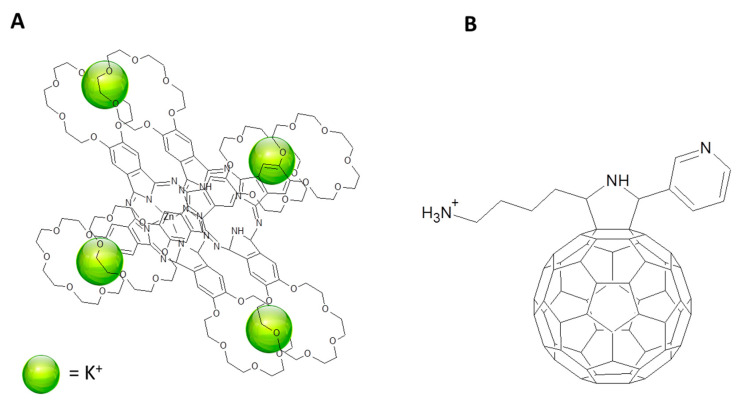
(**A**) ZnPcs derivative sandwich formed by the action of K^+^. (**B**) C60 derivative used to form the triad with (**A**) according to [97].

A more complex system was assembled by Fukuzumi, D’Souza and co-workers to mimic a bacterial photoreaction center: a supramolecular assembly of two ZnPcs in co-facial configuration and a fullerene derivative was proposed [96]. ZnPc was functionalized with 4 crown ether units (see Figure 10A) and a sandwich-type structure was obtained by the action of K^+^ (Figure 14A). C_60_ brings two chemical groups that are in charge to bind the phthalocyanines: a pyridine group able to coordinate the ZnPc central atom and an alkyl ammonium cation that electrostatically interacts with the crown ether group (Figure 14). Then, a supramolecular assembly formed by two co-facial phthalocyanines and two fullerenes (one on each side of the Pc/4K^+^/Pc sandwich) is obtained. Charge separated state lifetime was measured in 6.7 µs and it is significantly longer than the charge separated state lifetime recorded for analogous assemblies [97].

Even though ZnPcs are the most used metallophthalocyanines for obtaining charge and energy transfer towards acceptor molecules, the central metal atom, as well as the substituent chemical groups [17], can be changed in order to modify the spectroscopic and electronic features of the macrocycle and exploiting different methods to realize supramolecular assemblies. For example, a fulleropyrrolidine that brings a catechol substituent is used to chelate the titanium central atom of a tert-butyl Ti(IV) phthalocyanine [98] and a charge-separation is photoinduced in the dyad. 

**Figure 15:**
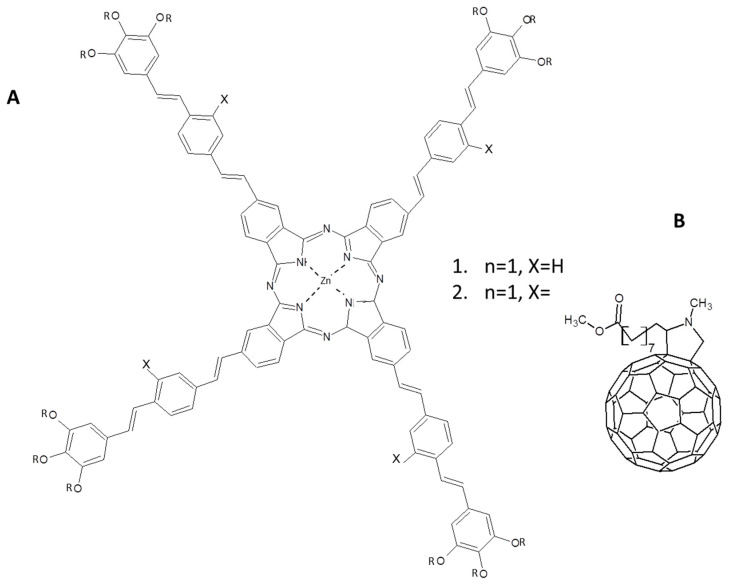
(**A**) Star-shaped zinc phthalocyanines and (**B**) fulleropyrrolidine derivative chemical structures used in [99].

Photoinduced charge transfer phenomena with very interesting applications for photovoltaic devices were recently studied in a supramolecular architecture formed by two differently functionalized ZnPcs reported in Figure 15 [99]. A mixture of compound **1** and **2** in ratio 1:1 exhibits very interesting properties both in CHCl_3_ solution and when spun-coated and annealed on solid support. The mixture of **1** and **2** in proper ratio is able to build up a supramolecular structure formed by a highly ordered liquid crystal donor-acceptor system. In particular, the conjugated substituents of the Pcs not only are crucial for the special aggregation of the superstructure, but they also represent the building blocks for obtaining an antenna system able to absorb energy from photons and to transfer it to the central part of ZnPc. In a cascade process, the energy transfer is followed by a charge transfer and separation toward fulleropyrrolidine derivative linked to the ZnPcs. 

Another case where covalently bonded fulleropyrrolidine derivative and ZnPcs form supramolecular aggregates with unique features is reported in [100]. The supramolecular formation of tubular structures of C_60_/ZnPc gives to the nanostructures interesting photo-induced properties. In fact, ultrafast charge separation (10^12^ s^−1^) and slow charge recombination (10^3^ s^−1^) processes characterize the supramolecular tubular aggregates. It is worth observing that the charge recombination mechanism for the supramolecular aggregates is deeply shifted in the Marcus inverted region. In fact, the lifetime of charge separated state appears six order of magnitude longer for the nanotubes aggregates than for the monomeric form of the covalent bounded C_60_/ZnPc dyad.

**Figure 16:**
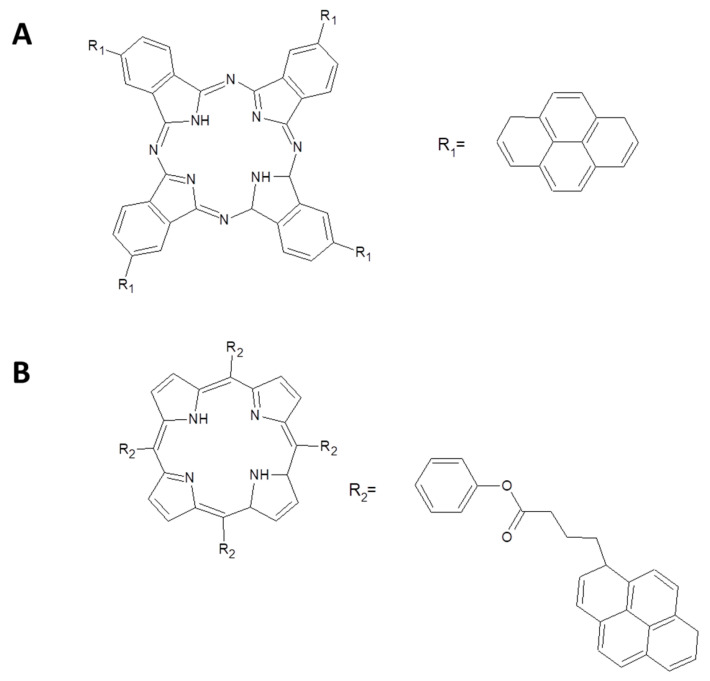
Structures of (**A**) phthalocyanine directly linked to four pyrene entities and (**B**) porphyrin linked to four entities of pyrene [101].

Another allotropic form of carbon, graphene, has been sometimes used as electron acceptor when coupled with phthalocyanine. As well as C_60_, graphene energetic levels are suitable for accepting electrons by phthalocyanine when the two species are adequately coupled. π-stacking interactions between graphene layer and the pyrene substituents appended both to phthalocyanine and porphyrins (Figure 16) ensure the formation of a supramolecular assembly porphyrin-graphene and phthalocyanine-graphene [101].

Photoinduced charge transfer are observed for both the systems with comparable charge separation and charge recombination rates (1.4 × 10^11^ s^−1^ and 5.2 × 10^9^ s^−1^, 3.5 × 10^12^ s^−1^ and 7.4 × 10^9^ s^−1^ for the porphyrin-graphene and Pc-graphene, respectively). 

In [102], the phthalocyanine is used as an electron donor in combination with Au-porphyrin in the role of the electron acceptor. Pyridyl substituents attached to the porphyrin have been used, as already reported for many pyridyl C_60_ derivatives, to coordinate the Zn central atom of tert-butyl phthalocyanine. The supramolecular complex appears stable enough (a binding constant of 2.94 × 10^4^ M^−1^ was measured). The electron transfer process from electron donor ZnPc to porphyrin moiety was evidenced by spectroscopic methods with a rapid charge separation process and charge recombination with a relatively slow rate (4.10 × 10^9^ s^−1^). 

A saddle distorted dodecaphenyl-substituted porphyrin was used, in supramolecular combination with a 8-times phenyl substituted ZnPc, as electron acceptor [103]. In particular, the saddle distortion of porphyrin macrocycle facilitates the protonation of pyrrole nitrogen atoms and it promotes the acceptor feature of this macrocycle and, in this particular case, it has been used to supramolecularly interact with carboxylate group of a 4-pyridincarboxylate (4-PyCOO^−^). Pyridin termination of 4-PyCOO^−^ was used to bind Zn atom of Pc and the supramolecular assembly has been obtained. By means of photophysical characterizations the authors evidenced that, under 410 nm photoexcitation, both porphyrin and phthalocyanine are excited. Then, the porphyrin transfers energy on Pc and a very efficient electron transfer takes place from Pc LUMO towards the dicationic porphyrin. 

**Figure 17:**
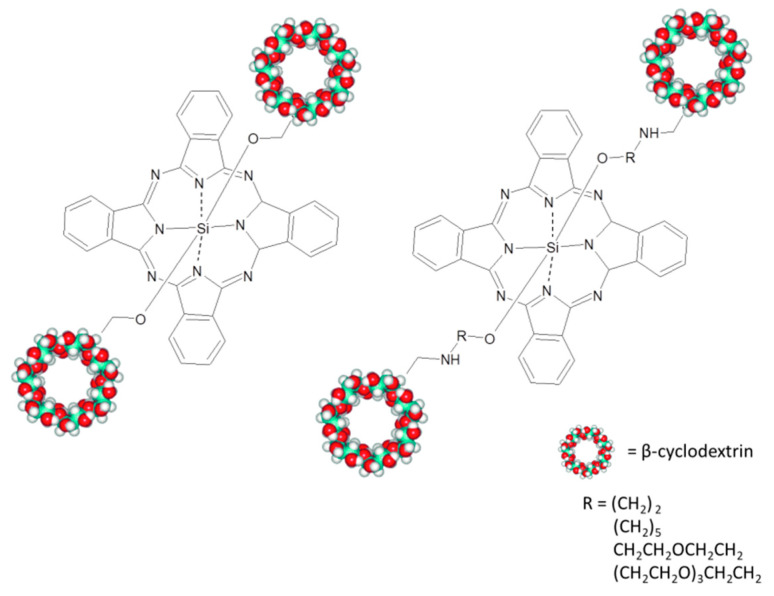
Silicon(IV) phthalocyanines axially substituted with permethylated β-cyclodextrin used in [104].

The same mechanism has been not only observed in [104] but tuned by changing the length of the ligand used to link porphyrin and phthalocyanine. Supramolecular assemblies were obtained using a tetrasulfonated porphyrin and several silicon(IV) phthalocyanines axially substituted with permethylated β-cyclodextrin (CD) (Figure 17) with different molecular bridges. As well as in the other porphyrins/Pcs systems, an energy transfer occurs from Pc to porphyrins and a charge transfer takes place from Pc to porphyrins. Both these mechanisms are strongly influenced by spatial conformation and length of the spacer. 

Same Pcs were used to form supramolecular triads in the presence of both porphyrins and a β–cyclodextrin conjugated subphthalocyanine [105]. The absorption bands of the three chromophores are well-separated and this allows to separately excite them. According with the author conclusions, it can be supposed that the triad is arranged as followed: subphthalocyanine moiety complexes the tetrasulfonated porphyrin and, on the other side, the phthalocyanine supramolecular binds the porphyrin derivative. When the subphthalocyanine is excited an energy transfer towards ZnPc is observed and the porphyrin derivative acts as a molecular bridge. Further energy transfer is observed from porphyrin to ZnPc when the porphyrin’s Soret band is excited. In addition to the energy transfer phenomenon, the same authors demonstrated that an electron transfer takes place in the supramolecular triad formed by β–cyclodextrin conjugated subphthalocyanine/tetrasulfonated porphyrin/permethylated β-cyclodextrin SiPc [106]. 

**Figure 18:**
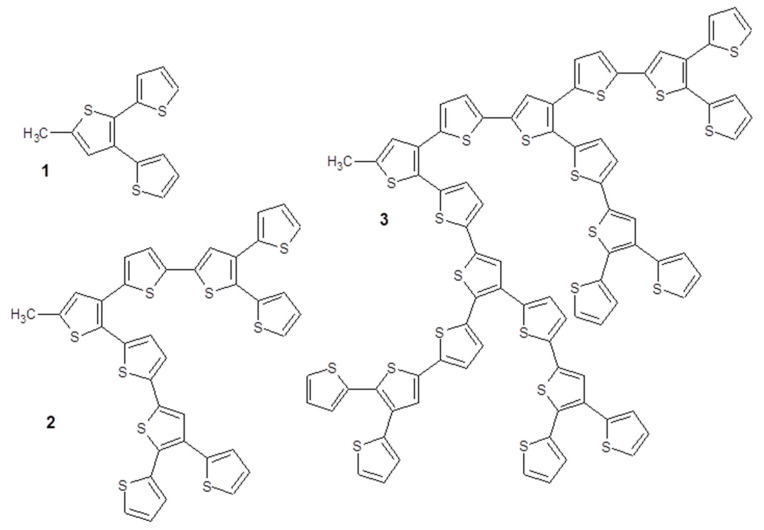
Molecular structures of the oligothiophene ligands used in [108].

With similar philosophy, a supramolecular dyad represented by a Zn anionic substituted phthalocyanine and a Zn cationic porphyrin derivative was used to modify a SnO_2_ electrode. The two macrocycles are photo-active in very large range of visible spectrum and the device obtained by the deposition of the electrostatically bounded porphyrin/Pc dyad on SnO_2_ showed an efficiency of 0.5% under AM 1.5 illumination [107]. Finally, ruthenium(II) phthalocyanine was coordinated by six different dendritic oligothiophene ligands (Figure 18) [108].

In this study, the energy and charge transfer phenomena were evaluated changing the solvent polarity and the dendritic oligothiophene, highlighting that polar solvents promote the charge transfer process while non-polar solvents induce an almost exclusive energy transfer. Energy transfer is the process that allows to obtain an IPCE% of about 5% in a supramolecular dyad formed by a pyridine-substituted sub-phthalocyanine that coordinates the Zn central atom of a zinc phthalocyanine derivative [109]. The supramolecular dyad is interfaced with TiO_2_ to build up a DSSC and short circuit current of 11.6 mA cm^−2^ and open circuit voltage of 589 mV are obtained when the photodevice is illuminated under a solar simulator. 

**Figure 19:**
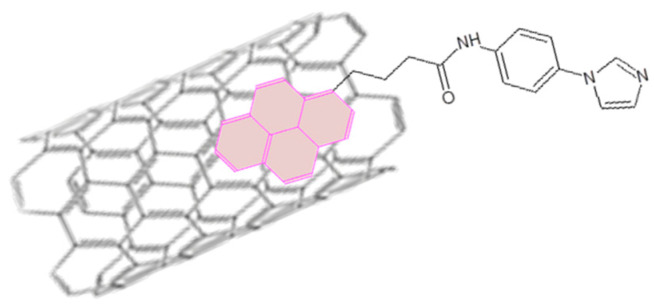
Interaction between CNT and pyrene functionalized with the imidazole entity according to the mechanism proposed in [113].

Few examples of (supra)molecular assemblies of Pcs and carbon nanotubes are reported. It is mainly due to the energetic HOMO–LUMO levels position and to their highly attitude to aggregate in all solvents. Nevertheless it is interesting to consider two different cases: in the first one the phthalocyanine derivatives play the role of electron donor towards the nanotubes [110], in the second case a phthalocyanine derivative is used in the uncommon role of electron acceptor [7]. In both cases the attitude of the carbon nanotubes to aggregate was overcome using the π-electron cloud of Pc that interacts with the π-electrons of CNTs enhancing their solubility. This approach is often preferred to the covalent functionalization of the carbon nanotubes since the covalent approach can induce important modifications to electronic and spectroscopic features of CNTs [39,111,112]. In an another system [113] ZnPc derivative was coupled with CNTs by means of the use of a pyrene (magenta colored in Figure 19) functionalized with the imidazole entity. π-π stacking between pyrene moiety and CNTs allows to solubilize the carbon nanotubes, furthermore the imidazole moiety is able, according to the several examples already reported, to coordinate Zn atom in the Pc derivative ring. The photoinduced charge transfer was evidenced from Pc derivative to CNTs throughout the pyrene-based bridge.

**Figure 20:**
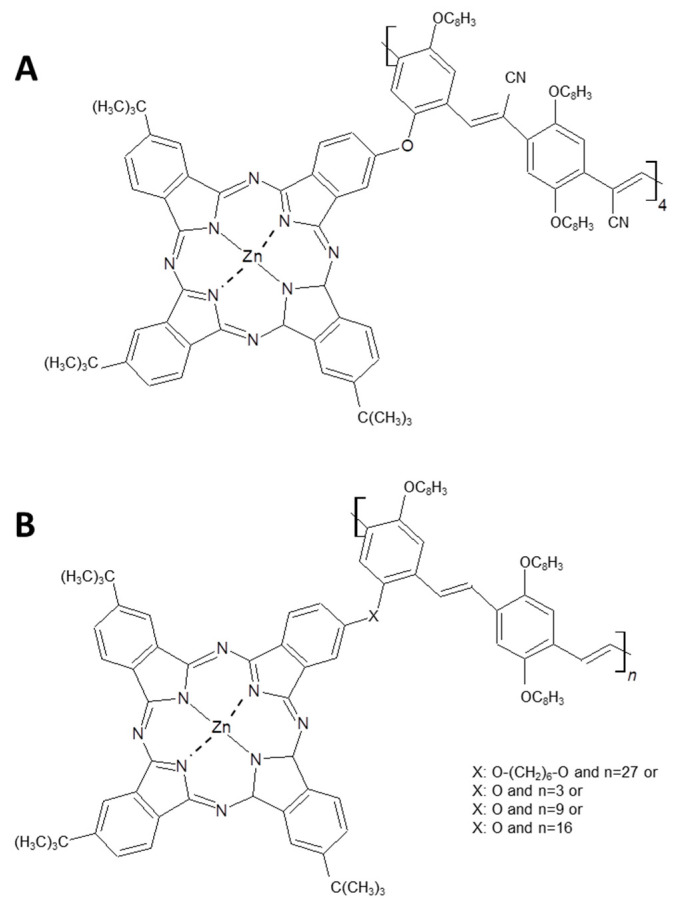
Six different ZnPcs chemical structures used to interact and to improve CNTs solubilization. ZnPc derivative (**A**) and ZnPc with X = O-(CH_2_)_6_-O and *n* = 27, reported in (**B**), appear particularly efficient in the SWCNTs solubilization [114].

Furthermore, the supramolecular functionalization of CNTs can be obtained by means of photo-active molecules or polymers as already reported for other cases of study. It allows to control the distance and the geometry of the supramolecular adduct. In particular, ZnPc laterally substituted with conjugated poly(*p*-phenylenevinylene) oligomers (Figure 20) were dispersed in presence of single walled carbon nanotubes (SWCNTs) in THF and in dimethylformaldehyde [114]. 

All the functionalized Pcs shown in Figure 20 allow to obtain very stable and highly concentrated single walled CNTs suspension. Fluorescence studies demonstrated that shortest oligomer (Figure 20A) and the Pc with the most flexible structure (X: O-(CH_2_)_6_-O and *n* = 27) better interact with SWCNTs and in both cases a charge transfer from the photoexcited ZnPc to SWCNTs was observed by time-resolved transient absorption measurements. 

**Figure 21:**
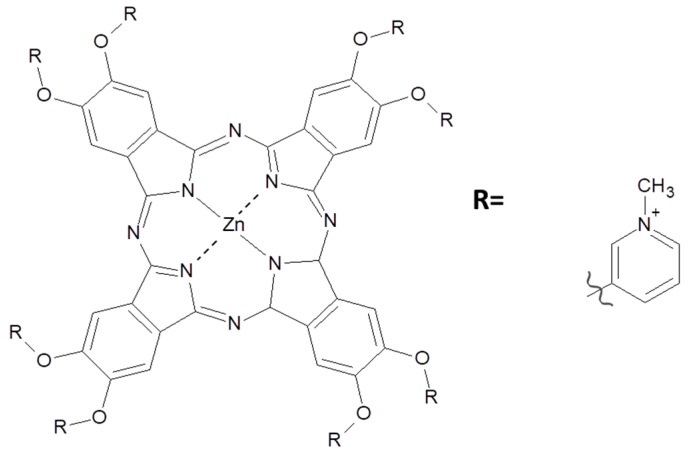
Cationic zinc phthalocyanines used to improve SWCNTs solubilization in water [7].

In order to confer electron acceptor features to a phthalocyanine, it needs to be functionalized with highly electron withdrawing substituents. On the other side, the suitability of SWCNTs as electron donors have been already reported when they are coupled with molecules such as fullerenes and perylenes [114,115]. A strategy to have simultaneously a Pc with electron acceptor characteristic and a phthalocyanine able to interact and dissolve SWCNTs in aqueous solution was proposed in [7]. In Figure 21 the chemical structure of the zinc phthalocyanine derivative used to suspend the nanotubes and to form the photoactive supramolecular dyad is presented. 

Spectro-electrochemical experiments were reported, confirming the photo-induced electron transfer from SWCNTs to Pcs and a solar device using the dyad ZnPc/SWCNTs dyad was carried out. Short circuit current and open circuit voltage value depend on the photoactive layer thickness with a maximum of 0.37 mA cm^−2^ and 16 mV, respectively. 

A case where the ZnPc is used as an energy acceptor was reported some years ago by Brühwiler and co-workers [116]. In the sake of truth, the aim the research was to explore the possibility to obtain a very efficient charge transfer from a dye molecule (thionine) to the ZnPc in a zeolite channel. The authors induced the absorption of ZnPc derivatives with different functionalizations in the zeolite. According to the Pc functionalization, the absorption process is ruled by Van der Waals, electrostatic or covalent bindings. Electrostatic forces appear more suitable to build the structure up and an energy transfer was photo-induced from thionine molecule to ZnPc by means of FRET along the zeolite channel. 

The possibility to use metallic and semiconducting nanoparticles was explored to enhance the photo-induced charge transfer and energy transfer phenomena in the presence of organic molecules [117], in particular dyes [118] and macrocycles like porphyrins and phthalocyanines, even though few examples are strictly connected to solar devices for converting photons in electric current. Gold nanoparticles were used to build up a supramolecular dyad with tert-butyl substituted ZnPc and a charge transfer in toluene solution between the two species was evidenced by spectrophotometric approaches [119]. Furthermore, the presence of the nanoparticles does not influence the formation of the supramolecular triad with PC_70_BM that shows interesting and efficient charge transfer phenomenon. 

## 4. Pcs-Based Supramolecular Assemblies for Photocatalyst Applications

**Figure 22:**
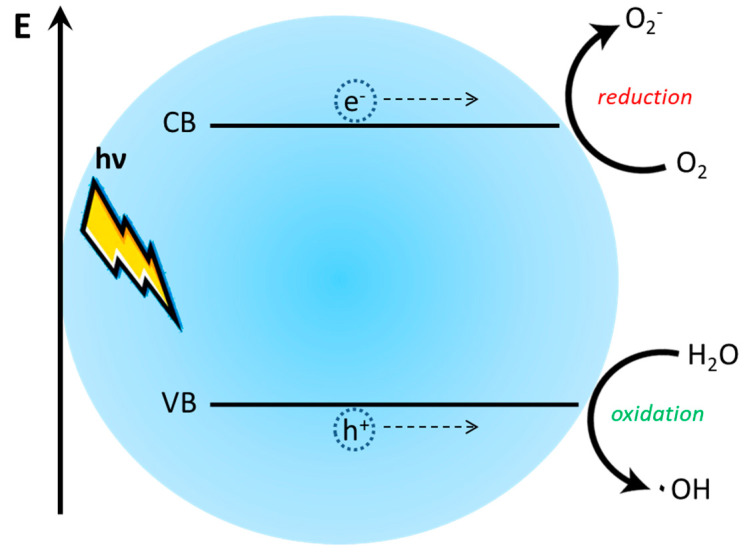
Schematization of the photo-induced formation of the radical O_2_^•−^ and the formation of the hydroxyl radical (OH^•^).

The mechanisms ruling the photodegradation and/or more in general the photocatalysis is very similar to the photo-generation of the charge-separated state in a photo-active material. As reported in the previous paragraph, the exciton is promoted by the absorption of a photon of appropriate energy, then the hole-electron photo-generated pair can be separated at the interface with an appropriate material. In a typical photo-catalytic process, an appropriate photon promotes the electron excitation from the HOMO level (or from the valence band) to the LUMO level (or to the conduction band) [120]. As well as in the case of a photovoltaic device, electron and hole migrate toward the catalyst surface to recombine or to promote a redox reaction with the compounds adsorbed on the catalyst. The positive hole reacts with water molecule promoting the formation of the hydroxyl radical (OH^•^) and the electron reduces the oxygen molecules adsorbed, forming the radical O2^•−^ (Figure 22) [121]. Superoxide and hydroxyl radicals are very reactive species that interact with interesting molecules inducing their modification. 

In light of this, it is easy to understand that many compounds and supramolecular architectures used in the photovoltaic devices are employed for obtaining efficient photocatalytic system and, as in the case of the photovoltaic devices, the supramolecular arrangement is crucial [122,123]. For example, in the present section, dyads formed by phthalocyanine derivatives and functionalized perylenes, as well as C_60_, porphyrins and carbon nanotubes will be examined. As a consequence, the strategies used to obtain the supramolecular dyads are, in many cases, very similar to those ones already examined. Therefore, analogously to the approach used in Section 3, we will start examining the photocatalytic systems based on Pcs/PDI derivatives.

**Figure 23:**
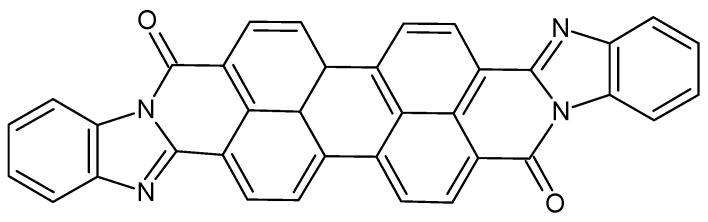
Chemical structures of the perylene derivative PDI used in [124].

The perylene derivative illustrated in Figure 23 was used in combination with unsubstituted free-based [124] and Co-metallated Pc [125]. It is interesting to observe that, even though the energetic levels of the two Pcs are very similar, no evolution of O_2_ has been observed in the case of the free-base Pc/PDI supramolecular assembly. On the contrary, when CoPc is used, an interaction between OH^−^ (in alkaline environment, pH = 11) and Co(III) can be predicted. So, under illumination, an electron transfer is induced from Pc to PDI. The hole-doped CoPc can efficiently activate OH^−^ for dioxygen evolution.

The supramolecular nature of the assembly obtained between a tetrasubstituted copper Pc (*p*-type organic semiconductor) and an asymmetrically substituted PDI (*n*-type) appears particularly important for the photocatalytic activity. The pyridine substituent was attached on the imide of a PDI derivative (PDI-P) promoting the formation of a hydrogen bonded supramolecular adduct with –COOH group of a 2,9,16,23-tetrakis(4′-carboxyphenoxy) phthalocyanine copper (CuTPc) after a simple procedure [126]. The photocatalytic performances of PDI-P/CuTPc supramolecular assembly were evaluated by monitoring the photodegradation of Rhodamine B (RhB) aqueous solutions under visible light illumination. It is interesting to observe that the RhB photodegradation efficiency is equal to 23% in the presence of CuTPc and 27% when CuTPc and PDI-P are simply mixed. On the contrary, when the supramolecular adduct PDI-P/CuTPc is obtained the efficiency increases up to 80%. In order to explain the photodegradation mechanism, the role of three different scavengers was tested, i.e., *p*-benzoquinone (used to quench the formation of superoxide radical ·O_2_^−^), KI (as hole quencher) and isopropanol for quenching the ·OH formation. The charge separation enhancement obtained when KI is added to the solution is an obvious consequence of the increased number of free electrons; *p*-benzoquinone does not influence the photocatalytic process efficiency and isopropanol almost completely quenches the RhB photodegradation, suggesting that the photocatalytic process is driven by the ·OH. Starting from these considerations, the following mechanism is proposed. As known, the electrons of both CuTPc and PDI-P can be excited on the respective LUMO levels when irradiated by visible light. The promoted electron on electronic excited level of the Pc derivative moves to the LUMO of PDI-P when reaches the interface between the *p*- and the *n*-semiconductor. The electrons on the PDI-P excited level react with O_2_ forming ·O_2_^−^ that induces the formation of ۰OH and then the RhB degradation takes place. On the other side, the electron role in the ·O_2_^−^ formation reduces the e^−^-h^+^ recombination rate, the holes can migrate from HOMO level of *n*-type molecule towards the HOMO level of CuTPc and directly reacts to degrade RhB [126]. Tetrasulfonated copper Pc was coupled to the 3,4,9,10-perylenetetracarboxylate [127]. As known, the energetic levels of the two organic species are suitable to form an electron donor/electron acceptor dyad and furthermore, in the particular case, the molecular dimensions of both the species perfectly fit if arranged in an edge-on configuration. It allowed to form a sandwich configuration between two layered double hydroxide (LDHs). Under visible illumination, electron transfer is promoted from cationic Pc to PDI derivative and the obtained holes can be used for the degradation of organic dyes. 

The well-known ability to obtain a charge separated specie in the presence of C_60_ (and derivatives) using Pc as electron donor was employed even to induce photodegradation of organic molecules. As already stressed in the previous paragraph, intermolecular arrangement plays a crucial role in the charge separation phenomenon. In fact, the efficiency of a composite nanoarchitecture obtained by means of AlPc chloride and C_60_ appeared two times higher than the simply mixture of AlPc and C_60_ in the CO_2_ production and triethylamine photodegradation [128]. The enhancement of photocatalytic features is a consequence of the interface optimization among the AlPc nanostructures and C_60_ nanoaggregates. It improves the charge separation process generating free charges available for interacting with organic molecules. An improvement of CO_2_ photogeneration was obtained by the substitution of CoPc to AlPc [129]. In fact, the same procedure was used to obtain CoPc/C_60_ nanoclusters and their photocatalytic activity appeared more performing than the one recorded using AlPc/C_60_ adduct when irradiated by a low power (∼1 mW cm^−2^) visible light source. Furthermore, the authors evidenced that the proposed dyad gave CO_2_ four times larger than that of well-known inorganic commercial WO_3_ when the systems were illuminated by 600 nm wavelength. A further confirmation of the most performing photocatalytic features of CoPc, if compared with other metallated Pcs, is given in [130] where the authors supramolecularly assembled dyads of Zn, Cu, Co and free-based Pcs (and porphyrins) with a bipyridylfulleropyrrolidine. The obtained adducts were used to decorate the titanium dioxide surface and the efficiency of phenol photocatalytic degradation was evaluated for each system. In the sake of the truth, the system that uses Co porphyrin as electron donor appeared the most performing one. Anyway, for all the adducts, an electron transfer under visible light stimulus from Pc (or porphyrin) to C_60_ derivative, and from the fulleropyrrolidine to TiO_2_ can be predicted. The injected electrons can react with the dissolved oxygen molecules promoting the formation of superoxide and hydroxyl radicals, very reactive species involved in the phenol degradation. 

**Figure 24:**
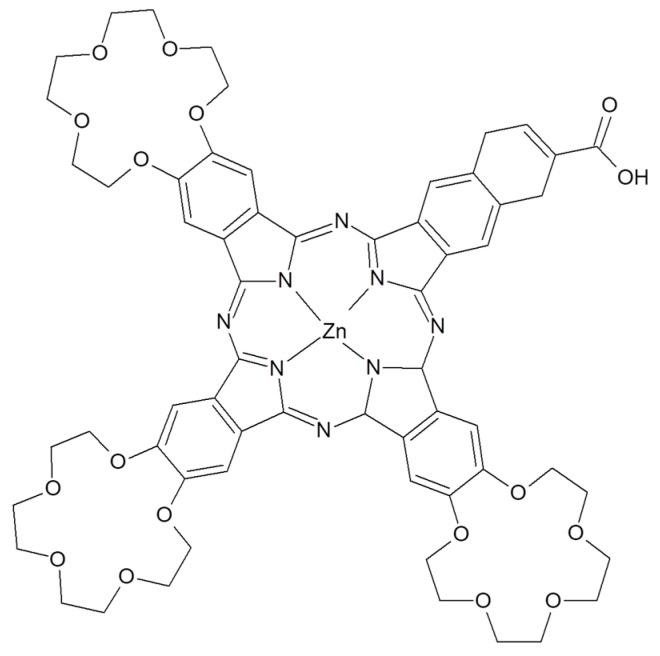
Chemical structure of ZnPc with three 15-crown-5 ether functional groups and one carboxyl group [134].

In the field of the photocatalysis, Pcs are often coupled with nanoparticles for several reasons: for example nanoparticles with magnetic features can be used to easily remove Pc catalyst [131], metallic plasmonic nanoparticles can work as plasmonic antennas to transfer energy on Pc catalyst [132] or phthalocyanines derivative can be used to shift the photocatalytic activity toward the visible range [133]. A ZnPc derivative functionalized with a carboxyl group and three 15-crown-5 ether substituents (Figure 24) was designed and used as a photosensitizer for a Pt-loaded graphitic carbon nitride for H_2_ photoproduction with visible illumination [134]. The –COOH substituent binds the ZnPc derivative on the Pt nanostructure in cooperation with π-π interaction of the delocalized electrons of Pc and the planar structure of the graphitic carbon nitride. The crown-ethers are used to complex alkaline ions, in particular the addition of K^+^ strongly improves the photoactivity for H_2_ production. 

In a recent system, Ni/NiO was decorated with Cobalt Pc and it was demonstrated that the photocatalytic reduction of CO_2_ of the supramolecular system appears enhanced if compared with Ni/NiO and CoPc separated components [135]. The chemical physical interpretation of this phenomenon is very similar to the one previously proposed for full organic systems, such as for example C_60_/Pc derivative adducts. Furthermore, the Ni/NiO nanosystem is able to perform photocatalytic reduction of CO_2_ when irradiated with an opportune wavelength and, simultaneously, it works as electron acceptor from CoPc.

## 5. Pcs-Based Supramolecular Assemblies for Photodynamic Therapy (PDT) Applications

**Figure 25:**
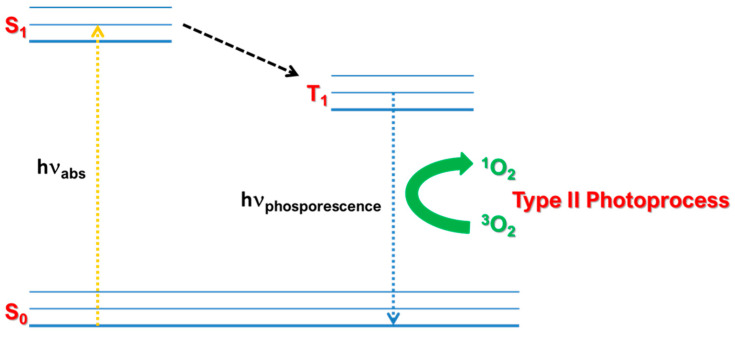
Schematized Jablonski’s diagram for a typical PS.

The peculiar physical chemical features of Pc derivatives make them suitable for PDT applications. PDT, in detail, is a therapeutic approach which ensures the inactivation of cancer cells thanks to the production of toxic singlet oxygen by employing photosensitizers (PS), as Pc molecules, porphyrins, perylene bisimide compounds, etc [59,136]. Basically, the PS absorbs light in the ground state, S_0_, promoting electrons to the excited state, S_1_. At this point, PS molecule could undergo the non-radiative process of Intersystem Crossing (ISC) generating the triplet excited state, T_1_ (Figure 25). Although ISC is a forbidden process requiring a spin inversion, good PS compounds are characterized by efficient ISC. In particular, from T_1_, PS could relax by phosphorescence decay or by spin exchange with another triplet state molecule [137], as molecular oxygen ^3^O_2_, which is in its natural triplet state, generating ^1^O_2_ by Type II Photoprocess (Figure 25) [138].

**Figure 26:**
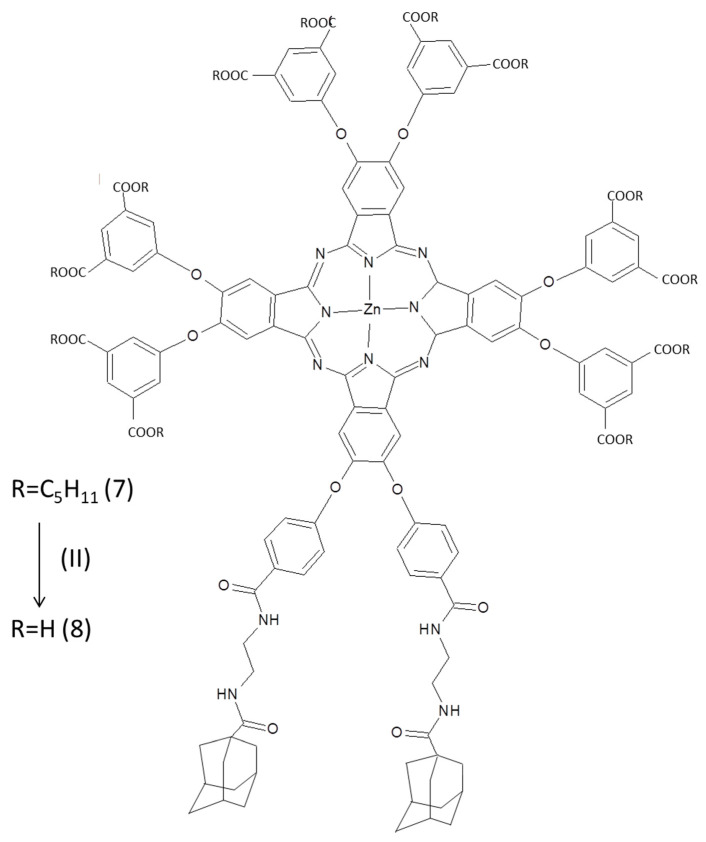
Octahoxosubstituted ZnPc bearing two adamantane groups [140].

A good PS is supposed to have several characteristics, i.e., appropriate light absorption features, rather in red spectral region (λ > 650 nm), low dark toxicity, photo-stability, high quantum yield of photogenerated ^1^O_2_ (Φ_Δ_) and relative long lifetime of triplet state [138]. Pcs are good PS taking in account such features; unfortunately they are well-known to be not water soluble, reducing the possibility for in vivo applications and increasing the risk of self-quenching phenomena due to the stacking events [138]. A possibility to overcome such a drawback is to supramolecular self-assemble Pcs moieties with cyclodextrins (CD) as proposed by Ravoo and collaborators [139]. A Pc derivative ad hoc functionalized by adamantane groups was supramolecularly assembled with cyclodextrin unilamellar vesicles (CDVs) made by modified β-CDs, improving singlet oxygen photogeneration in aqueous environment. They developed the synthetic route to obtain both an amphiphilic β-CDs with alkyl chains on the secondary face and oligoethylene glycol units on the primary face and an octaoxosubstituted ZnPc bearing two adamantane groups (Figure 26). The obtained amphiphilic β-CDs were reported to self-assemble into CDVs [140,141].

**Figure 27:**
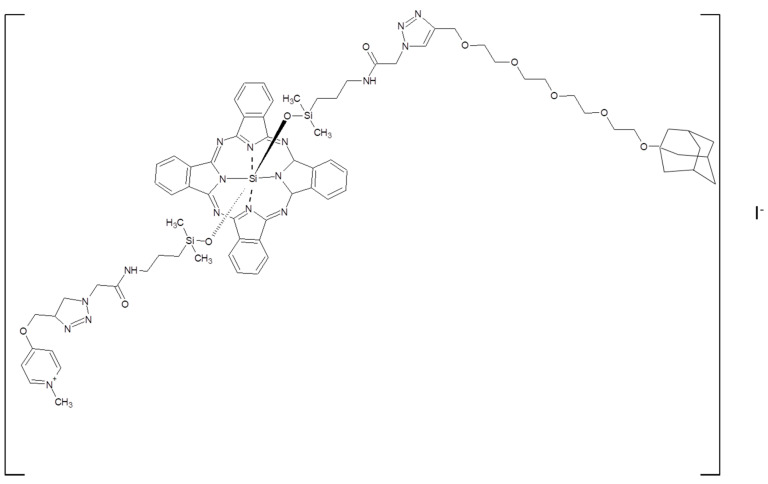
Chemical structure of Si(IV)Pc derivative modified with an axial pyridinium and an axial adamantane moiety used in ref [146].

The adsorption of ZnPc moieties on CDV surface was supposed to be driven by the adamantane groups, according to the literature [142,143] and was supported by the drop to more negative value of the ζ–potential measured: the presence of several free carboxylic groups of ZnPc contributes to lower such a value. Dynamic light scattering (DLS) measurements demonstrated that bare CDVs and ZnPC-CDVs supramolecular assembly diameters were comparable (about 100 nm), suggesting that in the presence of ZnPc the strong electrostatic repulsion among CDVs ensured to avoid aggregation phenomena in aqueous media. This is particularly interesting for the photophysical features of the proposed supramolecular assembly. In fact, absorption spectra of aqueous solution of ZnPc and ZnPc-CDVs were recorded and showed the typical bands of a Pc derivative in both cases [144]. It is worth highlighting that the shoulder located at about 620 nm, mainly due to Pc aggregates [139], disappears in the ZnPc-CDVs spectrum, suggesting that the supramolecular assembly permits to preserve the monomeric form of the ZnPc derivative in aqueous matrices. Even, the emission features were similar and in good agreement with the literature [145]. Then, the fluorescence and the singlet oxygen quantum yield (Φ_F_ and Φ_Δ_) were evaluated and between ZnPc and ZnPc-CDVs aqueous solution interesting differences were underlined. Φ_F_ was enhanced by more than twice and Φ_Δ_ resulted four times increased in the adduct. Basically, the supramolecular ZnPc-CDVs adduct prepared was characterized by enhanced ^1^O_2_ photogeneration properties thanks to the monomerization of the ZnPc derivative and, presumably, by good biocompatibility features thanks to the presence of the CD, so can be thought for PDT applications. Starting from these observations, Strassert and collaborators (2016) optimized a supramolecular system based on such CDVs and an asymmetric Si(IV)Pc derivative ad hoc modified (Figure 27) with both an axial pyridinium and an axial adamantane moiety in order to achieve a higher control on the behavior in aqueous matrices and on the interaction with CDVs surface [146].

**Figure 28:**
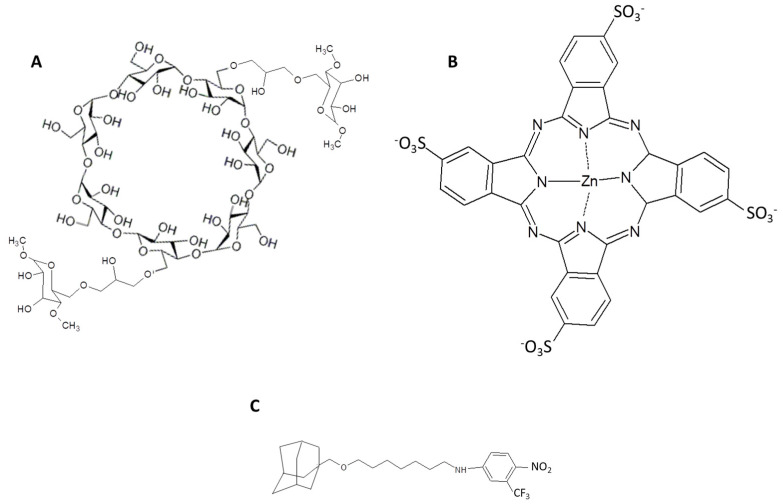
Chemical structures of poly-β-cyclodextrin (**A**), zinc phthalocyaninetetrasulfonate (**B**) and adamantylnitroaniline (**C**) derivatives [151].

The positively charged pyridinium bearing axial substituent was expected to improve water solubility and the adamantane bearing chain was exploited for the adsorption of the Pc onto the surface of the CDVs due to their reported affinity [147]. Further, SiPc were reported to have some advantages for PDT applications if compared with ZnPc mainly related to higher Φ_F_ and Φ_Δ_, thanks to a reduced tendency to aggregation [148]. The photophysical features of SiPc in dichloromethane (DCM), in water and in water in the presence of an increasing concentration of CDVs were compared. SiPc is present as monomer with interesting Φ_F_ and Φ_Δ_ value in DCM and in presence of CDVs upon supramolecular assembly formation and was organized in J aggregates when it is dissolved in water. Then, they calculated the binding constant exploiting the Langmuir fitting of the fluorescence (at 675 nm) titration at increasing CDVs molar concentration from 0.02 mM to 0.2 mM finding a value of 33 × 10^−3^ M^−1^ [149]. This value is in good agreement with a host-guest interaction between the adamantane group and the vesicles surface, considering some additional interactions between the positive charged pyridinium moieties and negative charged CDVs surface and some hydrophobic-hydrophobic interactions among SiPc and CDVs bilayer [150]. The system was tested on a methicillin resistant *Staphylococcus aureus* strain (MRSA) and no dark toxicity was detected for bare SiPc and SiPc-CDVs. Instead, upon illumination, both systems induced relevant bacterial inactivation in 150 min. This could mean that J aggregates are still useful for singlet oxygen photogeneration but also that the binding to the bacteria surface could somehow induce the monomer formation. Nonetheless, the fact that the supramolecular adduct worked as well can represent a good starting point for biological applications in order to have the possibility to introduce another therapeutic agent, for a bimodal approach, or some targeting molecules to deliver the assembly in a specific site by using CDVs as delivery systems. In the context of a bimodal therapeutic approach, based on CDs and a Pc derivative as photosensitizers, Sortino and collaborators developed a supramolecular assembly for combining the production of singlet oxygen and nitric oxide under light stimulus [151]. The system was based on a tetrasulfonate ZnPc derivative, an epichlorohydrin-β-CD copolymer and an adamantyl functionalized NO photodonor (Figure 28). The chosen copolymer was expected to form in water nanoparticles of about 25 nm of diameter thanks to the formation of glyceryl cross-linkers among β-CD units, characterized by high biocompatibility. The folded polymer configuration is characterized by the presence of hydrophobic nanodomains [152,153] which ensured the encapsulation of the ZnPc derivative through the benzene ring. The supramolecular interaction of the first aromatic ring induces further interactions in a cooperative way [151]. Absorption and emission spectra of aqueous solution of ZnPc and of ZnPc in presence of increasing concentration of the epichlorohydrin-β-CD network were obtained. The Pc derivative tends to form aggregates and not to fluoresce in water (becoming no active as PS), whilst, upon encapsulation, the monomerization was induced restoring red light emission. So, the obtained supramolecular system was also proposed for two photon excitation (TPE) fluorescence microscopy applications permitting to monitor the uptake by cancer cells. 

The system epichlorohydrin-β-CD/tetrasulfonate ZnPc was demonstrated to be characterized by high binding constant value in a 1:1 stoichiometry suggesting that the benzenesulfonate moieties can interact with the 3D CDs network due to the high density of cavity favoring the binding as monomer of the Pc, fundamental requirement for the ^1^O_2_ photogeneration and for TPE applications. Then, the chosen NO donor, characterized by an adamantyl moiety linked to a nitroaniline commercial derivative, was encapsulated in the CDs exploiting the well-known affinity of the adamantane for CDs [147], avoiding the internalization of the entire chromophore. The tilted configuration of the nitro group with respect to the aromatic ring plane guarantees the NO photorelease and this binding mechanism is able to prevent from the planarization [59]. The final assembly was characterized and, upon 532 nm excitation, both the ZnPc transient triplet state decay at 500 nm and the ^1^O_2_ phosphorescence decay at 1270 nm were monitored confirming the great potential of this hybrid nanostructured assembly. Moreover, NO photogeneration was confirmed by means of an amperometric sensor. The cellular internalization was checked by TPE imaging in vitro on an epidermal cancer cell line (A431) and, even, ex vivo on human skin. The cell viability was strongly reduced by the combination of photoreleased NO and ^1^O_2_, demonstrating that the three component system has very interesting features for this kind of applications.

In the same perspective, the same tetrasulfonate ZnPc was encapsulated by using a β-cyclodextrin-based polymer [154], which is reported to assemble in water into 30 nm nanoparticles [153]. As in the previous example [151], the encapsulation restored both the red light emission and the transient spectrum of the triplet state of the PS, suggesting the presence of the monomer. Indeed, the 680 nm absorption band was restored as well [155]. Even in this case, the system was thought for a bimodal approach further encapsulating a green emitting NO photodonor, preserving the photoactivation of this compound. The developed hybrid system was tested in vitro on a melanoma cells line both as platform for the monitoring of the internalization by means of Confocal Laser microscopy and as a platform to kill cancer cells upon light irradiation. Moreover, in the above frame, the tetrasulfonate ZnPc and the NO photodonor were encapsulated in a supramolecular assembly based on the β-cyclodextrin-based polymer and a dextran modified with alkyl side chain into a photoreleasing NO and ^1^O_2_ high biocompatible hydrogel [156].

Another proposed approach based on supramolecular assembly of natural polymer to exploit singlet oxygen production by Pc derivatives dealt with the use of nanocellulose crystals (CNC) [41]. CNC were obtained by a sulphuric acid hydrolysis procedure which permits to have negative charged CNC due to the presence of sulfate groups [157]. In this way, two cationic Pcs bearing eight pyridinium moieties were electrostatically linked to CNC surface. In particular, one of the two used derivatives was quaternized by methoxy(triethylenoxy) chains and presents tert-butylphenyl substituents at the 5-position of the pyridinium units. Basically, this second derivative was less water soluble than the first one. The obtained supramolecular adducts were demonstrated to have higher photo-antimicrobial activity due to singlet oxygen production with respect to bare Pcs. It is worth noting that suspensions of both the adducts in phosphate saline buffer (pH 7.4) actually were no more able to photorelease ^1^O_2_, due to strong aggregation phenomena. Nonetheless, once the biocompatible systems were placed in contact with Gram-negative and Gram-positive bacteria and a pathological yeast, thanks to the supramolecular interaction, the Pcs could be supposed to be released from CNC restoring ^1^O_2_ production. In fact, the photo-antimicrobial activity resulted improved for these supramolecular adducts if compared with porphyrin-based systems, but covalently linked [158,159].

**Figure 29:**
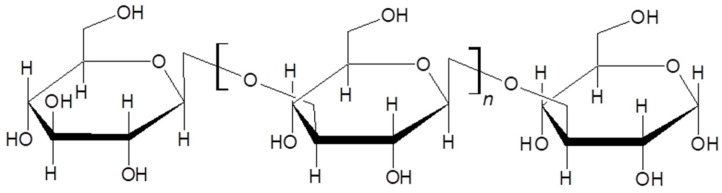
Curdlan chemical structure [161].

In the same frame of biopolymers supramolecular assemblies for Pcs delivery in PDT applications, curdlan (CUR) based nanoparticles were even proposed with interesting results [160]. CUR (Figure 29) is a β-(d)-glucan polymer able to spontaneously assemble in aqueous media into right-handed triple helices (t-CUR) [161]. The possibility to interconvert t-CUR into single strand CUR (s-CUR) in dimethyl sulfoxide (DMSO) was exploited to encapsulate a Pc derivative, creating CUR-Pc based nanoparticles of about 760 nm with a red-shifted Q band located at about 752 nm. There is a great result for biomedical applications, due to the deeper penetration of tissues guaranteed by the red region of the light spectrum. CUR-Pc systems were tested on HeLa cells showing no dark toxicity and interesting capability to kill cancer cells upon long-wavelength light irradiation. 

**Figure 30:**
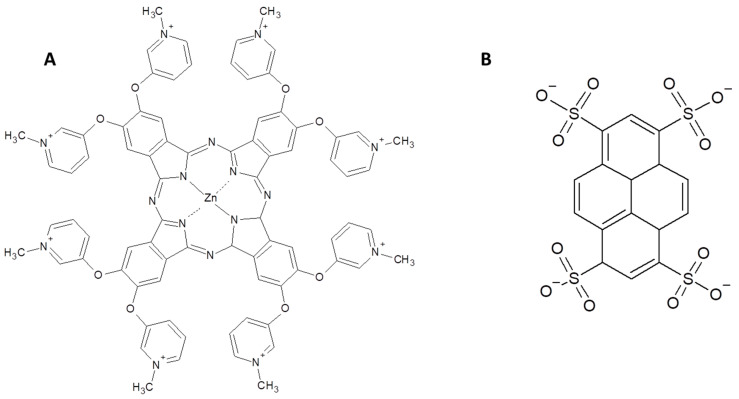
(**A**) Octacationic ZnPc derivative (octakis(1-methyl-3-pyridiniumoxy)-ZnPc) [51] and (**B**) tetra-anionic pyrene derivative (1,3,6,8-pyrenetetrasulfonic acid) [170].

Another particularly interesting approach to develop bio-inspired hybrids for PDT applications based on Pc derivative is the employment of virus capsid proteins allowing to obtain a platform able to deliver Pcs derivative and to preserve the photoproduction of ^1^O_2_ [162,163,164]. Virus like particles (VLP) produced from the coat protein (CP) of cowpea chlorotic mottle virus (CCMV) are reported as versatile nanosystems for drug delivery applications [165,166,167]. CCMV is a single-strand RNA plant virus characterized by a capsid of ca. 28 nm diameter, where CP are supramolecularly organized in 90 dimers in a icosahedral conformation [168]. Basically, CP can be released, disassembling the virus, and re-assembled, once RNA is separated, into VLP encapsulating chemical species, such as PS moieties. Tuning the composition (i.e., ionic strength) and the pH of the medium, different conformation of VLP can be obtained [163]. In this way, Cornelissen, Torres and collaborators were able to encapsulate a tetrasulfonate ZnPc in VLP as π-π stacked dimers [164]. The ZnPc dimers loaded VLP cellular internalization was checked inside RAW264.7 macrophage cells and the red light irradiation was confirmed to induce cell death [164] underlining the good potential of such systems. The supramolecular formation of ZnPc dimers nanosphere inside VLP was even theorized [163]. By changing the route to re-assemble VLP in presence of the Pc derivative, an important hypsochromic shift from 635 nm (typical of dimer configuration [169]) to 613 nm of ZnPc absorption band was recorded, suggesting the H aggregation of the molecules inside the VLP. Cryo-TEM images of the obtained ZnPc loaded VLP suggested the presence of high ZnPc density with the molecules organized as empty nanosphere in the inner part of the particle. Indeed, protein cages can thought as building blocks to develop systems encapsulating molecules with low water solubility [170]. Among these proteins, also apoferritin (aFt) from *Pyrococcus furiosus* was reported as an interesting biological system to be loaded for biomedical applications able to organize into hierarchical supramolecular assemblies [162]. aFt was mixed with a supramolecular assembly based on an octacationic ZnPc derivative (octakis(1-methyl-3-pyridiniumoxy)-ZnPc) and a tetra-anionic pyrene derivative (1,3,6,8-pyrenetetrasulfonic acid), reported in Figure 30 [51]. The π-π stacking between ZnPc and pyrene drove the assembly formation in a 1:1 stoichiometry. An important red shift of ZnPc Q band from 635 nm to 680 nm suggested the formation of the monomeric form of ZnPc upon pyrene complexation. This configuration would have preserved 4 positive charges on the ZnPc-pyrene supramolecular adduct. The electrostatic interactions allowed a hierarchical self-assembly process with the formation of ternary (ZnPc-pyrene-aFt) face-centered cubic (fcc) packed crystals.

This ternary complex was characterized in terms of Φ_Δ_ and represents the first example of crystalline supramolecular adduct preserving the photosensitizing features of the embedded PS [51]. 

Always in order to improve Pc water solubility and delivery in biological media, Kataoka and collaborators [171,172,173] reported about the possibility to synthesize dendrimers encapsulating Pc, as photoactive centre, and bearing ionic peripheral groups. Such dendrimeric moieties, represented by poly(benzyl ether) dendrons, were demonstrated to form supramolecular polyionic complex micelles (PIC) through electrostatic interactions with oppositely charged copolymers [174,175].

**Figure 31:**
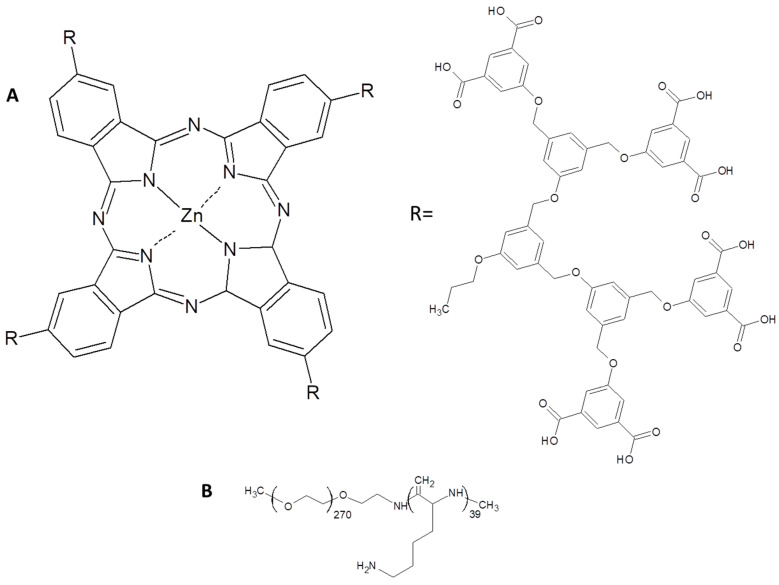
(**A**) dendrimeric ZnPc; (**B**) PEG–PLL used in [171].

Figure 31 shows the chemical structure of the employed anionic dendrimeric ZnPc (panel A) and of poly(ethylene glycol)–poly(l-lysine) (PEG–PLL, panel B) used as copolymer to obtain PIC. 

The dendrimers encapsulating Pc (DPc) based PIC, thanks to the steric hindrance of the dendrimer chains, were characterized by reduced ZnPc aggregation restoring fluorescent emission and singlet oxygen photo-generation, even if a hypsochromic shift of ZnPc peculiar absorption band from 685 nm to 630 nm was observed [173]. In particular, DPc micelles photo-cytotoxicity against HeLa cells was tested and compared to free DPc and it was found that, after 60 min irradiation, PIC were remarkably more toxic than DPc. Basically, free DPc had better performance as photosensitizing agent in terms of ^1^O_2_ production, but cellular internalization process seemed to be improved by the micelles. The system was indeed tested in vitro against A459 lung cancer cells [172] and the time-dependent morphological changes during PIC uptake were checked suggesting a unique pathway mediated by endocytosis. Micelles reach mitochondria inducing a huge photo-damage. 

Another kind of micelles reported to enhance ZnPc properties for PDT applications was obtained thanks to supramolecular assembly of sodium deoxycholate (SDC) and D-α-tocopherol acid polyethylene glycol succinate (TPGS) in specific molar ratios in order ensure the encapsulation of ZnPc in the hydrophobic core [176]. By changing SDC amounts, different mixed micelles morphologies were obtained, i.e spherical and rod-shaped. All the micelles were characterized by high Pc loading efficiency, good colloidal stability in physiological conditions, good Φ_Δ_ and remarkable ability of ZnPc release during the time, an important feature in a drug delivery system. The light-induced cytotoxicity of ZnPc loaded mixed micelles was investigated on A549 cells with interesting results. Also Poly(*N*-vinylpyrrolidone) (PVP) was mixed with Pc derivatives in order to obtain micelles [177]. 

**Figure 32:**
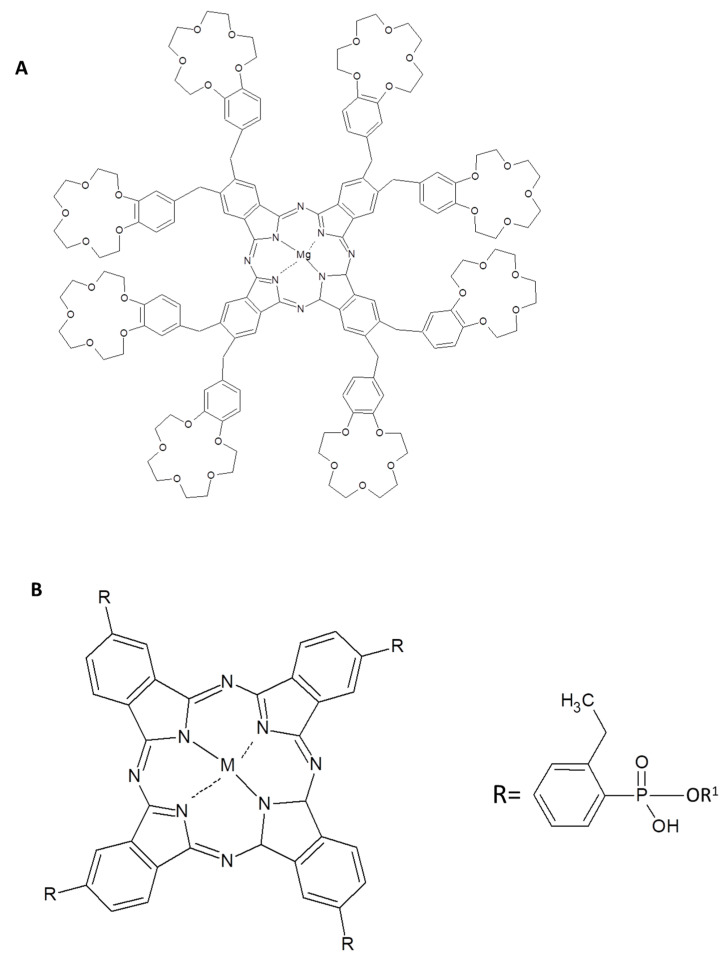
(**A**) crown-ether and (**B**) phosphoryl-containing derivatives [177].

In particular, crown-ether Pc and different phosphoryl containing Pc (Figure 32A,B respectively) were tested and the role of the metal ion to induce the monomer formation in presence of PVP in the case of the phosphoryl-containing Pc was underlined. Crown-ether derivative aggregation, instead, was not suppressed even with PVP, but slightly reduced by further SDC addition. Nonetheless, the crown-ether derivative based supramolecular assembly was demonstrated to be cytotoxic in vitro against HeLa cells and to reach the perinuclear region. The phosphoryl-containing ZnPc, instead, remained in the cell periphery.

Moreover, nanoparticles assembled by mixing a ZnPc derivative (4-sulfonatophenoxy-substituted ZnPc) and different azobenzene amphiphiles were reported as very appealing nanoplatform for antibacterial PDT [178]. 

**Figure 33:**
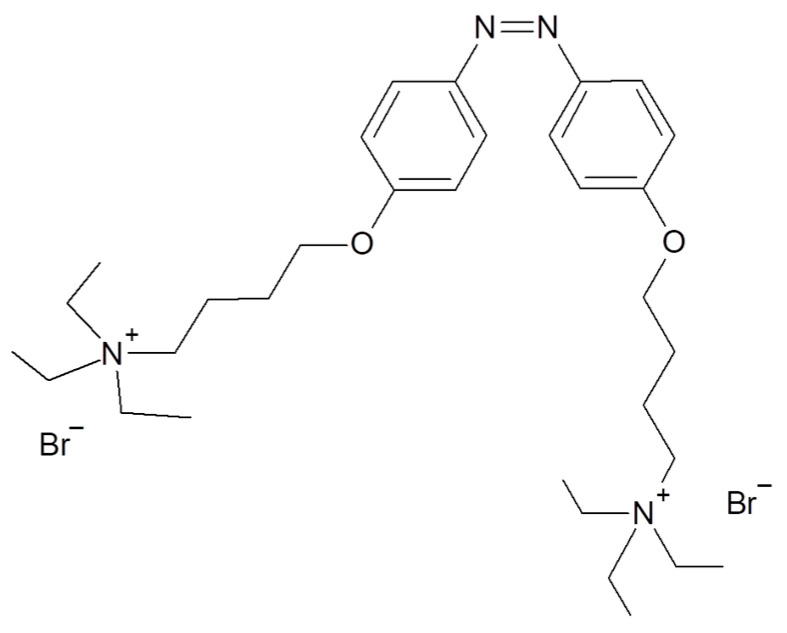
Schematic structure of compound Azo 1 [178].

In particular, the compound Azo 1 (Figure 33) was found to induce the greatest change in the UV-Vis spectrum of ZnPc suggesting a strong host-guest interaction mediated by π-π stacking phenomena, as even confirmed by the recorded fluorescence quenching upon nanoparticles formation. 

It is worth highlighting that thanks to the superior features of azobenzene, the nano-assembly can be assembled and disassembled by using light as external trigger [179], locking and unlocking the ZnPc in a form able to photogenerate singlet oxygen or not. Upon 1 min UV irradiation (365 nm), nanoparticles were disassembled and became able to kill bacterial cells, in particular *E. coli* and *S. aureus*, under red light irradiation.

Another reported approach to overcome Pc drawbacks as PS agent envisaged the chemical modification of Pc derivatives by developing novel synthetic routes and in order to tune Pc molecules aggregation and supramolecular organization [180,181]. Huang and collaborators [180] synthetized two ZnPc derivatives, a tetra- and a di-α-substituted ZnPc. A triethylene glycol (TEG) chain was used to form a hybrid aza-/oxa-crown-substituted phthalocyanine. The two derivatives displayed interesting self-assembly and photophysical features. Crown ether-substituted Pc, in fact, are known to self-assemble into π-π columnar aggregates which could lead to liquid-crystalline phases, highly ordered thin films or a gel [182]. The substitution induces a strong red shift of Q band to 699 nm in the case of the tetrasubstituted Pc and to 685 nm for the di-substituted Pc. Both molecules are characterized by good Φ_Δ_ values, especially the di-substituted one (0.74) calculated in dimethyl formamide. The induced J aggregation of the two Pc molecules was deeply investigated in CHCl_3_ and CH_2_Cl_2_ in presence of different metal ions demonstrating that singlet oxygen production reduced, if J aggregation took place. Finally, the cytotoxicity and the cellular uptake tests carried out on HepG2 human hepatocarcinoma cells clearly indicated the di-α-substituted ZnPc was characterized by good properties as PS. More recently, Zengin and collaborators [181] reported the synthesis of a novel ball-type ZnPc. This derivative showed an interesting Φ_Δ_ value of 0.89, much higher than the value reported for the un-substituted ZnPc but, unfortunately, the investigations were performed only in DMSO.

**Figure 34:**
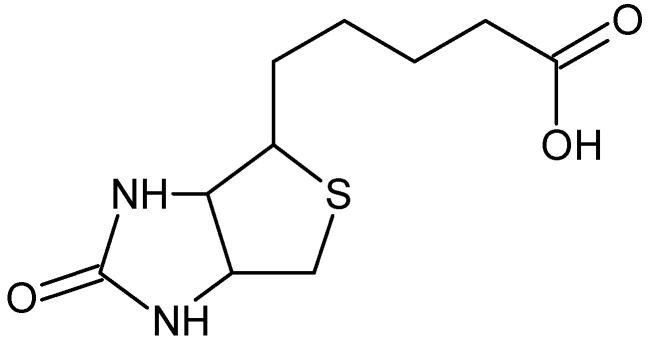
Biotin chemical structure [185].

The possibility to develop nano-assemblies of photosensitizers with a switchable design was even investigated [183,184]. In particular, regarding Pc molecules, a ZnPc derivative and a SiPc derivative were further modified in order to bear biotin (Figure 34) moieties allowing the “turn on” of Pc photoactivity specifically in the tumor site. Biotin, in fact, is a growth cell promoter and the demand for biotin in cancer tissues is abnormal. So, biotin receptor is overexpressed in these cells and can be used to specifically internalize biotin based supramolecular assemblies [185]. 

Basically, a ZnPc derivative was modified with triethylene glycol (TEG) linker (Pc-TEG and the hydroxyl group of TEG was further functionalized with biotin [184]. 4 different derivatives were obtained: Pc-1-TEG (with one TEG unit), Pc-2-TEG (with 2 TEG units), Pc-4-TEG (with 4 units) and Pc-4-TEG-B (with 4 TEG units bearing biotin). The first two derivatives did not self-assemble into nanostructures in water, whilst Pc-4-TEG and Pc-4-TEG-B formed spherical nanoparticles. Pc-4-TEG-B, in particular, was demonstrated to form high stable nanoparticles of size ranging between 100 and 200 nm (NanoPcTB). Photophysical characterization of NanoPcTB in water and of Pc-4-TEG-B monomer in DMSO, clearly, indicated that the assembly quenched the fluorescent emission of the embedded ZnPc, which resulted strongly aggregated with an important blue shift of the Q band in the absorption spectrum. The possibility to restore the photoactivity of NanoPcTB was tested in vitro by adding increasing amount of avidin, a biotin-specific binding protein. The system disassembled, thanks to the biotin-avidin interaction, allowing the generation of singlet oxygen. The addition of biotin, instead, permitted to re-assemble the nanoparticles, confirming the switchable behavior. NanoPcTB were tested in vivo in A549 tumor (biotin receptor positive) bearing mice by means of fluorescence imaging, confirming the restoring of the fluorescent emission even if in the starting solution was totally quenched. Furthermore, the cell viability assays on A549 and HeLa cells (both positive for biotin receptor) confirmed the outstanding potential of the system. Analogously, an amino-modified SiPc was functionalized with biotin moieties [183], and in presence of different amount of Cremophor EL (CEL) used as surfactant in water, nanoparticles with narrow size distribution of 10, 20, 40 and 90 nm (NanoPc10, NanoPc20, NanoPc40, NanoPc90) were obtained. By increasing the size of the NanoPc, the fluorescent emission quenching was stronger, but the addition, in vitro, of avidin, again, allowed to restore the photoactivity even for NanoPc90. Cytotoxicity assays and intracellular fluorescence imaging performed on HepG2 cells confirmed the “switch on” of the nanoparticles both in the case of NanoPc20 and NanoPc90. Nonetheless, NanoPc20 was revealed to be much more toxic of NanoPc90 due to mitochondria localization highlighted by in vivo fluorescence imaging upon NanoPc administration via tail in mice bearing H22 murine hepatocellular tumors. 

**Figure 35:**
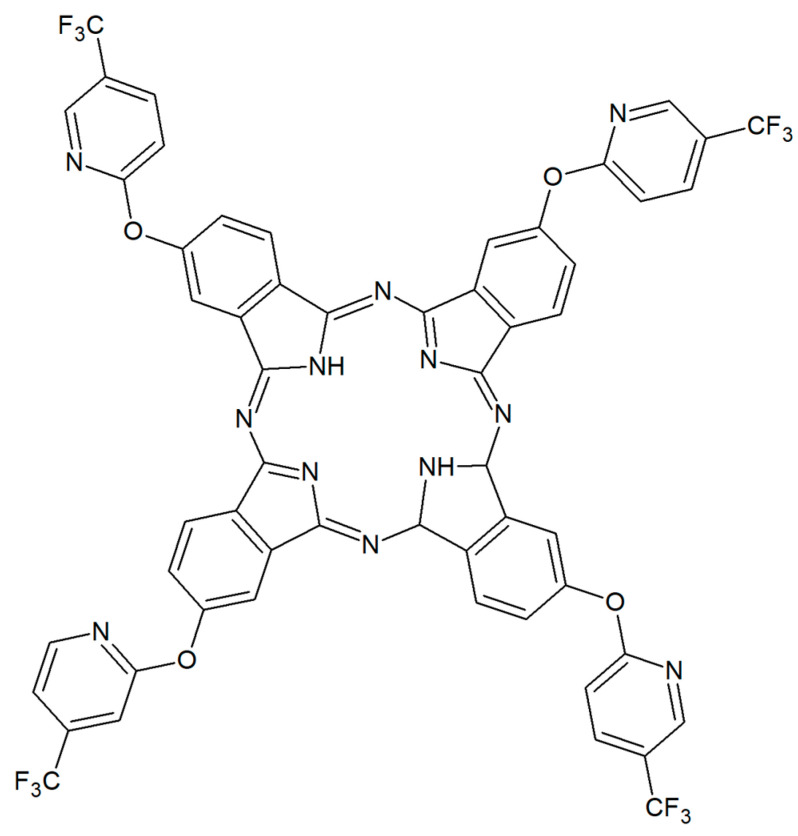
Chemical structure of the free base 4-((5-(trifluoromethyl)pyridin-2-yl)oxy)phthalocyanine utilized to assemble the hybrid structures with GQDs in the ref. [191].

Pc derivatives were also super-assembled with carbon based materials in order to obtain hybrid materials with peculiar photophysical characteristics [186,187]. Carbon based materials, such as carbon dots, graphene quantum dots, carbon nanotubes, have attracting always more interest due to their unique properties that make them suitable for several applications including bio-imaging, sensing, photocatalysis, drug delivery [188,189,190]. Hybrid materials based on graphene quantum dots and Pc derivatives are reported as interesting nanoplatforms with higher triplet state quantum yield Φ_T_, longer fluorescence lifetime and enhanced ^1^O_2_ photogeneration if compared to bare Pc derivatives. Nyokong and collaborators developed a one-step hydrothermal synthesis route of GQDs in presence of ascorbic acid and the Pc derivative reported in Figure 35, both the free base and the metallated one (Zn) [191]. The system was assembled by π-π interactions, which did not allow to observe the monomeric form of the free base Pc in the supramolecular nanostructures.

Anyway, the two obtained hybrid materials were characterized, in aqueous media, and in both cases the triplet quantum yield increased, underlining that GQDs support intersystem crossing from excited singlet to triplet state. This promoted, of course, an enhancement in the production of singlet oxygen. Further, Nyokong and collaborators, in another work, proposed ad hoc modified GQDs with BODIPY moieties to tune the interaction with a ZnPc derivative by means again of π-π interactions [187]. Even in this case, the supramolecular adduct was characterized by improved singlet oxygen generating ability, fundamental characteristic for the developing of PDT systems. 

In another approach, a polyfluoroalkyl substituted SiPc derivative (SiPc-F) was synthetized and used to assemble a switchable hybrid system with single walled carbon nanotubes [186]. The supramolecular assembly was built up exploiting, first, host-guest interactions among SiPc-F and pyrene decorated β-CD (*p*-β-CD), synthetized according to the literature [192] and supposed to bear two pyrene moieties each CD. Such an interaction was confirmed by the red shift of SiPc-F Q band from 683 nm to 730 nm. Then, pyrene substituents were exploited to drive π-π interactions with CNTs sidewalls, generating the supramolecular complex. In this case, the presence of CNTs had a dual role. Thanks to the photothermal capability, which can be used as therapeutic approach [193], the hybrid system was able to induce a localized temperature enhancement: upon 12 min irradiation at 680 nm (400 mW cm^−2^) the temperature reached 50 °C. CNTs alone did not show the same temperature increase. Simultaneously, the temperature increase induced probably both the thermal expansion of CNTs in the hybrid system and a change in the chemical equilibrium of the complex with the consequent release “light-stimulated” of SiPc-F. Fluorescence emission of SiPC-F was quenched in the complex by CNTs, so its release restored the fluorescence and the possibility to generate singlet oxygen. The antibacterial properties were checked against *E. coli* and the hybrid system showed good results in an aqueous solution containing 10% of DMSO. The higher toxicity of the assembly if compared with SiPc-F alone or SWNTs could be attributed to the synergic effect of photothermal and photodynamic effect. 

In the context of developing supramolecular assembly that can exploit synergic effect of more than one component, supramolecular nanovehicles (SNVs) were assembled encapsulating a tetrasulfonate ZnPc derivative, as PDT agent, and doxorubicin (DOX), as chemotherapeutic agent, into an ad hoc designed copolymer, folic acid chemically-modified polyvinylpyrrolidone-b-polyacrylic acid (PVP-b-PAA-g-FA) [194]. Folic acid receptor is overexpressed in several cancer cells and folic acid can be thought as a specific target molecule [195]. The developed superior supramolecular assembly ensured synergistic chemotherapy-PDT targeted cancer imaging and therapy. The copolymer had the key role to ensure the formation of micelles like structures in presence of DOX; in fact, acidic DOX groups partially neutralize the ionic folic acid chains guaranteeing the formation of an inner hydrophobic core. The remaining ionized folic acid moieties combining with PVP allows the formation of an external shell exposed to the hydrophilic environment. SNVs, so, had an amphiphilic behavior. ZnPc, then, was internalized in such a shell by hydrogen bonds formation with PVP terminations [196]. The obtained SNVs ensured to have ZnPc in monomeric form optimizing ^1^O_2_ production in situ. The system was tested in vitro on HepG2 cells and in vivo in HepG2 tumor-bearing mice by intravenous injection into the tails. The synergistic effect confirmed higher anticancer activity than that of each single component of the assembly with a very low drug dose of 1 mg kg^−1^ considering both the dose of ZnPC and the dose of DOX. 

Also, the possibility to super-assemble nanoparticles of Pc derivative with mitoxantrone was investigated [197]. Mitoxantrone (MA) is a anthracenedione anticancer agent characterized by a conjugated aromatic ring system and two amine groups. These chemical characteristics are important to permit π−π stacking events and metal−ligand interactions in the complex formation with Pc derivatives. An anionic ZnPc was chosen to drive the formation of such an assembly, and, by UV-Vis characterization, a 1:1 stoichiometry of the complex was found with a binding constant of K = (4.3 ± 0.8) × 10^5^. Morphological characterization allowed to find nanoparticles of about 60 nm. Other drugs were tested, including DOX, but did not permit to form nanoparticles so well-dispersed as MA with the selected ZnPc. In the assembly, ZnPc photo-activity is quenched by the formation of H aggregates, but the supramolecular nature of the interaction allowed to disassemble the nanoparticles. In fact, MA anticancer activity was reported to be due to MA intercalation into DNA. This means that MA affinity towards DNA (binding constant is about 10^5^–10^6^) could be used to release ZnPc upon DNA competition with the interactions between ZnPc and MA. The switchable behavior of the system was tested in vitro by adding nucleic acid in solution and, indeed, at increasing concentration of DNA, ZnPc fluorescence was found to increase restoring singlet oxygen generation. In this way, promising results in vitro and in vivo were obtained by investigating the anticancer activity on MCF-7 cells and MCF-7 tumors bearing mice. 

## 6. Conclusions

Pc derivatives are organic compounds with intriguing physical chemical characteristics which make them suitable for different kinds of applications, especially when their peculiar photophysical features are concerned. Consequently, several examples of Pc molecules employed for driving photo-induced charge and energy transfer phenomena, singlet oxygen and other radical species photo-production, fluorescent sensing devices are reported in the literature. In particular, the possibility to supramolecularly assemble together different Pc derivatives and/or also with other species, such as other molecules, carbon allotropes, nanovesicles, etc., has been deeply investigated in this context. We, indeed, focused in the peculiar mechanisms that rule the above listed photoinduced phenomena when Pcs are supramolecularly arranged. This approach allows to build up even complex molecular architectures for selective analyte sensing based on specific aggregation states. Moreover, optimized charge and/or energy transfers for realizing photo-voltaic devices and photo-catalytic systems have been achieved and explored in this contribution underlining the advantages of the non-covalent interactions. Analogously, singlet oxygen generation has been preserved by the explored supramolecular assemblies guaranteeing in most cases the realization of interesting on-off systems, characteristic that can be almost exclusively reached upon supramolecular approach. 

## Figures and Tables

**Table 1:** Checked interfering elements used to evaluate the device selectivity. Adapted from ref. [55].

Foreign Substances	Maximum Concentration (ng mL^−1^)	Relative Error Caused (%)
Ba^2+^(Cl^−^)	500	−4.1
Mn^2+^(Cl^−^)	200	−2.2
Fe^3+^(Cl^−^)	200	−5.0
Mg^2+^(Cl^−^)	100	−3.1
Zn^2+^(Cl^−^)	50	−4.8
Cd^2+^(Cl^−^)	50	−4.1
Ca^2+^(Cl^−^)	50	−4.2
Hg^2+^(Cl^−^)	30	−3.9
Co^2+^(Cl^−^)	15	−4.5
dl-α-Aminopropionic acid	8000	+4.8
l-Phenylalanine	2000	−4.5
Glysine	2000	−4.6
l-Lyrosine	2000	+3.4
l-Arginine	2000	−4.9
γ-IgG	500	−3.6
HAS	200	+4.9
BSA	200	−4.8
CTAB	400	−5.0
SDBS	400	−1.7
Triton X-100	400	−0.2

ctDNA: 50 ng mL^−1.^.
